# Modulating the Energy Band Structure of the Mg-Doped Sr_0.5_Pr_0.5_Fe_0.2_Mg_0.2_Ti_0.6_O_3−δ_ Electrolyte with Boosted Ionic Conductivity and Electrochemical Performance for Solid Oxide Fuel Cells

**DOI:** 10.1021/acsami.2c06565

**Published:** 2022-09-19

**Authors:** Sajid Rauf, Muhammad Bilal Hanif, Naveed Mushtaq, Zuhra Tayyab, Nasir Ali, M. A. K. Yousaf Shah, Martin Motola, Adil Saleem, Muhammad Imran Asghar, Rashid Iqbal, Changping Yang, Wei Xu

**Affiliations:** † College of Electronics and Information Engineering, Shenzhen University, Shenzhen, Guangdong Province 518000, China; ‡ Hubei Collaborative Innovation Center for Advanced Organic Chemical Materials, Faculty of Physics and Electronic Science, Hubei University, Wuhan, Hubei 430062, P. R. China; § Energy Storage Joint Research Center, School of Energy and Environment, Southeast University, No.2 Si Pai Lou, Nanjing 210096, P. R. China; ∥ Department of Inorganic Chemistry, Faculty of Natural Sciences, Comenius University in Bratislava, Bratislava 84215, Slovakia; ⊥ Zhejiang Province Key Laboratory of Quantum Technology and Devices and Department of Physics and State Key Laboratory of Silicon Materials, Zhejiang University, Hangzhou 310027, People’s Republic of China; # College of Physics and Optoelectronic Engineering, Shenzhen University, Shenzhen 518060, China; ¶ New Energy Technologies Group, Department of Applied Physics, Aalto University School of Science, Espoo FI-00076 Aalto, Finland; ∇ Institute for Advanced Study, Shenzhen University, Shenzhen, Guangdong 518060, China

**Keywords:** LT-SOFC, Sr_0.5_Pr_0.5_Fe_0.2_Mg_0.2_Ti_0.6_O_3−δ_ electrolyte, Mg doping, high ionic conductivity, core−shell structure, energy band alignment

## Abstract

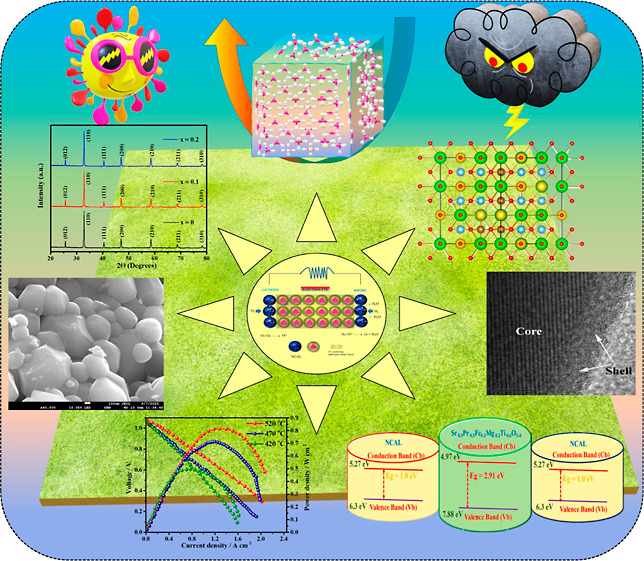

Achieving fast ionic conductivity in the electrolyte at low operating temperatures while maintaining the stable and high electrochemical performance of solid oxide fuel cells (SOFCs) is challenging. Herein, we propose a new type of electrolyte based on perovskite Sr_0.5_Pr_0.5_Fe_0.4_Ti_0.6_O_3−δ_ for low-temperature SOFCs. The ionic conducting behavior of the electrolyte is modulated using Mg doping, and three different Sr_0.5_Pr_0.5_Fe_0.4–*x*_Mg_*x*_Ti_0.6_O_3−δ_ (*x* = 0, 0.1, and 0.2) samples are prepared. The synthesized Sr_0.5_Pr_0.5_Fe_0.2_Mg_0.2_Ti_0.6_O_3−δ_ (SPFMg_0.2_T) proved to be an optimal electrolyte material, exhibiting a high ionic conductivity of 0.133 S cm^–1^ along with an attractive fuel cell performance of 0.83 W cm^–2^ at 520 °C. We proved that a proper amount of Mg doping (20%) contributes to the creation of an adequate number of oxygen vacancies, which facilitates the fast transport of the oxide ions. Considering its rapid oxide ion transport, the prepared SPFMg_0.2_T presented heterostructure characteristics in the form of an insulating core and superionic conduction via surface layers. In addition, the effect of Mg doping is intensively investigated to tune the band structure for the transport of charged species. Meanwhile, the concept of energy band alignment is employed to interpret the working principle of the proposed electrolyte. Moreover, the density functional theory is utilized to determine the perovskite structures of SrTiO_3−δ_ and Sr_0.5_Pr_0.5_Fe_0.4–*x*_Mg_*x*_Ti_0.6_O_3−δ_ (*x* = 0, 0.1, and 0.2) and their electronic states. Further, the SPFMg_0.2_T with 20% Mg doping exhibited low dissociation energy, which ensures the fast and high ionic conduction in the electrolyte. Inclusively, Sr_0.5_Pr_0.5_Fe_0.4_Ti_0.6_O_3−δ_ is a promising electrolyte for SOFCs, and its performance can be efficiently boosted via Mg doping to modulate the energy band structure.

## 1 Introduction

The fundamental obstacle in developing low-temperature solid oxide fuel cells (LT-SOFCs) is a lack of access to sufficient ionic conduction.^1,2^ The solid electrolyte is widely acknowledged to play a critical role in implementing the fuel cell function in SOFCs.^1,2^ For instance, 8% Y_2_O_3_-stabilized ZrO_2_ (YSZ) has been utilized as a promising ionic conductor at temperatures over 800 °C; however, the high operating temperature hinders its practical application for the real SOFC technology.^3^ The high operational temperatures (>800 °C) slow down the device startup/shutdown cycle, increasing the material cost/degradation rate and thus degrading the fuel cell’s overall performance. Nowadays, the decrease of the operating SOFC temperature is a major challenge that, however, possesses a major drawback. A substantial decrease in ionic conduction and an increase in Ohmic losses due to the reduction of the operating temperature are present.^2^ Various techniques have been adopted to address these challenges, including new materials as alternative electrolytes and fabricating ultra-thin electrolytes with a small Ohmic resistance by thin-film technologies.^4−9^ Nevertheless, adapting the thin-film technology to existing materials incurs high costs and negatively impacts stack fuel cell long-term stability.^10^ As a result of this shortage, tremendous efforts have been made to develop novel materials that overcome the aforementioned challenges while simultaneously providing a high ionic conduction (>0.1 S cm^–1^) at low temperatures (<600 °C).^2,11^

In the last few decades, the SOFC community has reported perovskite semiconductors in the form of electrodes, for example, MNO_3_ (M = Ba, Sr, Ca, Sm, La, etc. and N = Ni, Mn, Co, Fe, etc.), La_0.3_Sr_0.7_Fe_0.7_Ti_0.3_O_3−δ_ (LSFT), and SrFeTiO_3−δ_-based materials.^12,13^ Nevertheless, they have been scarcely reported as an electrolyte material to replace the pure ionic conducting electrolyte and integrate the ionic conduction behavior into semiconducting cathode materials utilized in SOFCs.^14^ Recently, the SOFC community reported semiconductors and their composites with high ionic conductivity obtained at low operating temperatures, the so called LT-SOFCs.^15,16^ The formation of the interfacial diffusion phenomenon due to semiconductor cathodes and their heterostructure has received tremendous attention; for example, Mushtaq et al. recently reported (SrFe_0.75_Ti_0.25_O_3−δ_–Sm_0.25_Ce_0.75_O_2−δ_) a semiconductor–ionic heterostructure material with a considerable ionic conductivity of 0.1 S cm^–1^ and a power density of 0.92 W cm^–2^) at 520 °C.^14^ Furthermore, the LaFe_0.65_Ti_0.35_O_3−δ_–Ce_0.8_Sm_0.05_Ca_0.15_O_2−δ_ heterostructure was reported with an appreciable power density of 0.98 W cm^–2^ and an ionic conductivity of 0.19 S cm^–1^ at 520 °C.^16^ In addition to these studies, the p–n heterojunction of the SrFe_0.2_T_0.8_O_3−δ_–ZnO electrolyte in the fuel cell device demonstrated a substantial power density of 0.65 W cm^–2^. It endorsed the reduction of electronic conduction with a significant ionic conductivity of 0.21 S cm^–1^ at 520 °C due to the creation of the p–n heterojunction.^17^ In the above studies, the interface played a vital role in enhancing the ion conduction to improve the electrochemical performance of the fuel cell device. Similarly, at the epitaxial Y_2_O_3_/ZrO_2_/SrTiO_3_(YSZ/STO) heterostructure interface.^18^ Compared to the simple planar YSZ structure, the YSZ/STO heterostructure demonstrated an 8 times magnitude enhancement in ionic conduction.

In addition, single-phase semiconductor electrolyte materials are also demonstrated to be promising functional electrolytes for LT-SOFCs. Recently, Zhou et al. reported a SmNiO_3−δ_ (SNO) perovskite semiconductor electrolyte with high proton conductivity and a power density of 0.225 W cm^–2^ at 500 °C due to the Mott transition effect in fuel cell conditions.^19^ Likewise, an electronic dominant semiconductor Li_*x*_Co_0.5_Al_0.5_O_2_ was utilized as an electrolyte where Li_*x*_Co_0.5_Al_0.5_O_2_ displayed an attractive protonic conductivity of 0.1 S cm^–1^, and a power density of 0.18 W cm^–2^ was achieved by introducing protons (H^+^) into the layered structure of Li_*x*_Co_0.5_Al_0.5_O_2_ at 500 °C.^20^ Generally, it was speculated that the fuel cell based on semiconductor electrolyte materials is prone to suffer from short-circuit problems due to the dominant electronic conduction. However, such materials showed a competent electrolyte role via various mechanisms, which are entirely different from conventional electrolytes’ working principle. For instance, the SNO electrolyte switched from electronic to insulator in the fuel cell operating conditions by virtue of the filling-controlled Mott transition to suppress its electronic conduction.^19^ Chen et al. reported the super ionic transport mechanism through the core–shell structure via insulating La–SrTiO_3−δ_ as an electrolyte with a high ionic conductivity of 0.221 S cm^–1^ at 550 °C in fuel cell conditions.^21^ Furthermore, Dong et al. demonstrated a pure TiO_2_ semiconductor as an electrolyte in a fuel cell system employing the energy band theory following the thumb rules. The purpose of employing the energy band theory is that TiO_2_ can work significantly as a functional electrolyte material and is reported in fuel cell devices with a high open-circuit voltage (OCV) and power density.^22^ Similarly, Rauf et al. have reported the semiconductor Tm-doped SrCeO_2−δ_ as an electrolyte in a fuel cell device and successfully employed the same rules of energy band theory with a higher OCV and power output.^23^ Moreover, the interesting Schottky junction effect has also been reported in semiconductor electrolyte fuel cells, which acted as a synergistic junction to restrict the flow of electrons from the anode toward the electrolyte and boost the ion conduction.^24^ At last, there is a promising semiconductor perovskite SrFeO_3−δ_, which has been rarely applied as an electrolyte; however, it possesses significant ionic conduction and exhibits stable redox activity of doped SrFeO_3−δ_. It can generate an enriched oxygen vacancy concentration via B-site doping of Ti.^13,25^

Inspired by the above studies, the single-phase semiconductors deserve more attention for further development and modification to explore their ionic conducting properties. The insulating characteristics of SrTiO_3−δ_ can be altered to a metallic behavior through suitable doping, which enable them to be utilized in solar cells, electronic devices, batteries, and fuel cell applications.^26^ The basis material of SPFT, perovskite SrFe_1–*x*_Ti_*x*_O_3−δ_ (SFT), has been applied to the electrolyte of SOFCs in the form of a semiconductor–ionic composite with SDC, which exhibited attractive ionic conductivity and fuel cell performance and indicated that SFT is a potential electrolyte.^14^ Moreover, considering the valuable characteristics introduced by Pr doping at the A site enhances the creation of oxygen vacancies.^27^ Therefore, we focus on Sr_0.5_Pr_0.5_Fe_0.4_Ti_0.6_O_3−δ_ (SPFT) to investigate its electrolyte functionality by enhancing the oxygen vacancies. Furthermore, the ionic characteristics are further modulated by the introduction of various concentrations of Mg doping into the B site of Sr_0.5_Pr_0.5_Fe_0.4–*x*_Mg_*x*_Ti_0.6_O_3−δ_ (*x* = 0, 0.1, and 0.2) to prepare three different samples with various compositions, such as Sr_0.5_Pr_0.5_Fe_0.4_Ti_0.6_O_3−δ_ (SPFT), Sr_0.5_Pr_0.5_Fe_0.3_Mg_0.1_Ti_0.6_O_3−δ_ (SPFMg_0.1_T), and Sr_0.5_Pr_0.5_Fe_0.2_Mg_0.2_Ti_0.6_O_3−δ_ (SPFMg_0.2_T). The modulation of these compositions is illustrated by the energy band structure and their crucial role in the transport of ions conduction and stoppage of electronic transport. Importantly, the doping of Mg ions at the B site of the perovskite semiconductor could generate oxygen vacancies, which overall enhances the ionic conductivity of SPFMg_0.2_T to a significant value of 0.133 S cm^–1^. Notably, the formation of the core–shell structure has provided a new mechanism for the oxide ion conduction at the surface layer of the insulating core SPFMg_0.2_T behaving as a heterostructured semiconductor core SPFMg_0.2_T with the superionic surface layer. Furthermore, theoretical calculations are performed to determine the density of states (DOS) of Sr_0.5_Pr_0.5_Fe_0.4–*x*_Mg_*x*_Ti_0.6_O_3−δ_ (*x* = 0, 0.1, and 0.2) compositions and to study the ground-state energy and the effect of Mg doping into SPFT to demonstrate their facilitation in the formation of oxygen vacancies. Based on all this mounting evidence, this research is expected to aid in developing single-phase semiconductors for use in fuel cell technology based on energy band engineering, as well as in understanding the working principle of fuel cells with different charge carriers.

## 2 Experimental Part Including Materials Synthesis and Methods

### 2.1 Materials Preparation and Characterizations

The hydrothermal technique assisted via the co-precipitation method is utilized to prepare Sr_0.5_Pr_0.5_Fe_0.4_Ti_0.6_O_3−δ_ and various Mg-doped compositions Sr_0.5_Pr_0.5_Fe_0.4–*x*_Mg_*x*_Ti_0.6_O_3−δ_ (*x* = 0.1–0.2). Sr(NO_3_)_2_, Pr(NO_3_)_2_·6H_2_O, Mg(NO_3_)_2_·6H_2_O, Fe(NO_3_)_3_·9H_2_O, and TiO_2_ from Sigma-Aldrich (purity 99.99%) were used as precursors without further purification. Ammonia (NH_3_), nitric acid (HNO_3_) (diluted 61%), and NH_3_^.^H_2_O were obtained from MACKLIN. Initially, the weighted amounts of Sr(NO_3_)_2_, Pr(NO_3_)_2_·6H_2_O, and Fe(NO_3_)_3_·9H_2_O were added into deionized water under continuous stirring to prepare Sr_0.5_Pr_0.5_Fe_0.2_Mg_0.2_Ti_0.6_O_3−δ_. In parallel, the weighted amount of TiO_2_ was dissolved in the appropriate volume of diluted nitric acid (HNO_3_) to form nitrates of TiO_2_ under vigorous stirring for 6 h at 60 °C. The obtained homogeneous solution of TiO_2_ nitrates was then put into the preceding solution and constantly agitated, while the pH was adjusted to 8.0 by using NH_3_·H_2_O. Moreover, the precipitating agent with a 1:2 ratio of metal cations and Na_2_CO_3_ was prepared in a solution. The precipitating agent was then added to the aforesaid solution to produce a bluish color, which was then transferred to the autoclave and put in the vacuum furnace for 6 h at 180 °C. The autoclave was then removed and allowed to cool at room temperature. The precipitate solution was then filtered and rinsed with ethanol and deionized water before being dried at 120 °C for 6 h. The well-dried materials were completely ground, then sintered at 1000 °C for 5.5 h at 3 °C/min, and then finely ground in an agate mortar. Electrochemical and physical characterizations were also performed for the as-synthesized powder. Similarly, the parent material SPFT and doped composition SPFMg_0.1_T were prepared via the same procedure to be utilized for further applications.

The phase structure of the synthesized Sr_0.5_Pr_0.5_Fe_0.4–*x*_Mg_*x*_Ti_0.6_O_3−δ_ (*x* = 0, 0.1, and 0.2) powders was investigated via X-ray diffraction (XRD, Germany, Bruker Corporation). MJAD 6.5 software was used to perform all XRD data refinement analyses. The surface morphology, particles distribution, and fuel cell device cross-sectional view of Sr_0.5_Pr_0.5_Fe_0.4–*x*_Mg_*x*_Ti_0.6_O_3−δ_ (*x* = 0, 0.1, and 0.2) were illustrated via field emission scanning electron microscopy (FE-SEM, MIRA3 TESCAN). Moreover, the microstructure of the various synthesized compositions of Sr_0.5_Pr_0.5_Fe_0.4–*x*_Mg_*x*_Ti_0.6_O_3−δ_ (*x* = 0, 0.1, and 0.2) powders was illustrated using a high resolution-transmission electron microscopy (HR-TEM) instrument (JEOL JEM-2100F) operated under an accelerating voltage of 200 kV. Energy-dispersive X-ray spectroscopy (EDS) was used to study the individual elemental mappings of the as-prepared materials, assisted by HR-TEM. X-ray photoelectron spectroscopy (XPS) using Al Kα radiation was used to investigate the developed materials’ surface charge transfer and chemical condition. The obtained XPS raw data were analyzed using CASA XPS software. In more detail, the energy structures of the parent and various doped compositions were analyzed by employing the UV–vis absorption spectroscopy (MIOSTECHPTY Ltd. UV3600 spectrometer) and ultraviolet photoelectron spectroscopy (UPS). UPS was used to calculate the valence band maxima under the unfiltered He–I (21.22 eV) gas discharge lamp and a total instrumental energy resolution of 100 meV. Electron energy loss spectroscopy (EELS) was employed to investigate the elemental mapping and the electron energy loss of the respective components of the treated Sr_0.5_Pr_0.5_Fe_0.2_Mg_0.2_Ti_0.6_O_3−δ_ after fuel cell performance evaluation.

### 2.2 Device Fabrication

There are three main fuel cell device fabrication components: anode, cathode, and electrolyte. As a result, SOFC devices with varied compositions based on Sr_0.5_Pr_0.5_Fe_0.4–*x*_Mg_*x*_Ti_0.6_O_3−δ_ (*x* = 0, 0.1, and 0.2) electrolytes were manufactured using a dry pressing process. The symmetrical electrodes were prepared using semiconductor NCAL-pasted Ni-foam (NCAL–Ni). At the same time, NCAL has recently been described as a catalyst capable of triple charge conduction (H^+^/O^2–^/e^–^) and strong catalytic activity in the hydrogen oxidation reaction (HOR) and oxygen reduction reaction (ORR).^24^ To prepare electrodes, a weighted amount of Ni_0.8_Co_0.15_Al_0.05_LiO_2_ (NCAL) powder [commercially obtained from Tianjin Bamo Company (TBC)] was mixed with the necessary volume of terpinol (a dissolving medium) to make a slurry. The slurry was painted on round-shaped nickel foam and desiccated at 120 °C for 0.45 h to form well-dried NCAL–Ni electrodes.

Furthermore, following the typical fuel cell device fabrication procedure, the single cell of SPFMg_0.2_T powder was compacted between the two sections of Ni–NCAL electrodes, distributed homogeneously under the pressure of 250 MPa to form a single cell. The cell’s configuration is Ni–NCAL/SPFMg_0.2_T/NCAL–Ni with the active area, thickness of the cell, and electrolyte thickness values of 0.64 cm^2^, 1.5 mm, and 500 μm, respectively. The cell is then coated with a silver paste as a current collector and also for gas sealing before being installed into the testing jig. Furthermore, the use of Ni-foam aids in ensuring the mechanical strength of the produced fuel cell device and supporting the electrode’s porous structure. Similarly, the other cells with different compositions were assembled with the same structure of Ni–NCAL/SPFMg_0.1_T/NCAL–Ni and Ni–NCAL/SPFT/NCAL–Ni in the same configuration for a comparative study. It should be noted that prior to electrochemical characterizations in terms of operation and performance measurement, each cell experienced online sintering at 600 °C for 1 h.

The single cell for gas chromatography–mass spectroscopy (GC–MS) characterizations was sealed in an Al_2_O_3_ tube using a Pt past and then Ceramabond 552-VFG sealant (Aremco) and heated to 650 °C.

### 2.3 Electrochemical Characterizations Tools

The electrochemical performance of the fuel cell device was evaluated in terms of current and voltage (*I*–*V*) and current and power density (*I*–*P*) characteristics. An electronic load IT8511 (ITECH Electrical Co., Ltd., China) was employed to record the data through software IT7000 at a scan speed of 0.02 A s^–1^ under a current–voltage sweep in the temperature range of 520–420 °C. The Gamry Reference 3000 electrochemical workstation (Gamry Instruments, USA) was used to perform electrochemical impedance spectroscopy (EIS) with flowing air (120–140 mL min^–1^) as an oxidant at the cathode during the process. Dry hydrogen gas served as a fuel with a flow rate of 120–150 mL min^–1^ to study the impedance and electrical properties of various components of fuel cell devices. Under OCV conditions, an AC voltage with an amplitude of 10 mV and a frequency ranging from 0.1 to 10^5^ Hz were applied. ZSimpwin was utilized for fitting the raw data using an appropriate equivalent circuit model to collect and simulate the raw data.

The gas product was determined by GC–MS (performed using a SCION GC–MS systems instrument of Bruker Corporation) in the temperature conducting detector (TCD) mode, which was employed to analyze the composition of the outlet gas measured at temperature 520 °C. A GC run was repeated every 10 min. The average value of three measurements was taken as the gas volumetric concentration for Faradaic efficiency calculation, and three average values are used for the plot. The flow rate of H_2_ was 30 mL min^–1^, and open air was used. The gas flow rate was determined using a flowmeter. The Faradaic efficiency was calculated as follows
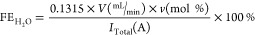
where *v* (vol %) is the concentration of H_2_O in the exhaust gas from the electrochemical cell (GC–MS data). *V* (mL/min) is the gas flow rate measured using a flow meter at the exit of the electrochemical cell at room temperature and ambient pressure.

### 2.4 Theoretical Studies via First-Principles Calculations

The density functional theory (DFT) and the first-principles calculations of Sr_0.5_Pr_0.5_Fe_0.6_Ti_0.4_O_3−δ_ and Mg-doped Sr_0.5_Pr_0.5_FeTiO_3−δ_ [Sr_0.5_Pr_0.5_Fe_0.4–*x*_Mg_*x*_Ti_0.6_O_3−δ_ (*x* = 0, 0.1, and 0.2)] perovskite oxides were performed using the Vienna ab initio simulation package (VASP) with the projector augmented wave (PAW) potential. The generalized gradient approximation (GGA) using the Perdew–Burke–Ernzerhof (PBE) function was used to model the exchange–correlation interaction between electrons. The plane wave cutoff energy is set to 520 eV. A 2 × 2 × 2 k-point grid Brillouin zone of the structure was used for calculations. All geometrical divisions were relaxed to the point where the energy changes were less than 1 × 10^–4^ eV and the forces on each ion were less than 0.01 eV/Å. The Hubbard-U term was added to the DFT (GGA) energy functional to characterize the 3d and 4f orbital structure of iron (Fe) and titanium (Ti). The values of *U*_eff_ = *U*–*J* were set to 6.31 and 5.0 eV for Ti and Fe, respectively.^28,29^ The oxygen vacancy formation energy of an oxygen vacancy was calculated from the energy difference between the total energy contained by the V_O_- and the sum of the total energy of the pristine and the chemical potential of an oxygen atom in an O_2_ molecule. The difference between the total energy stored by the V_O_- and the sum of the chemical potential of the oxygen atom in an O_2_ molecule and the total energy of the pristine was used to determine oxygen vacancy formation energy.

## 3 Results and Discussion

### 3.1 Structure and Morphology

**Figure 1:**
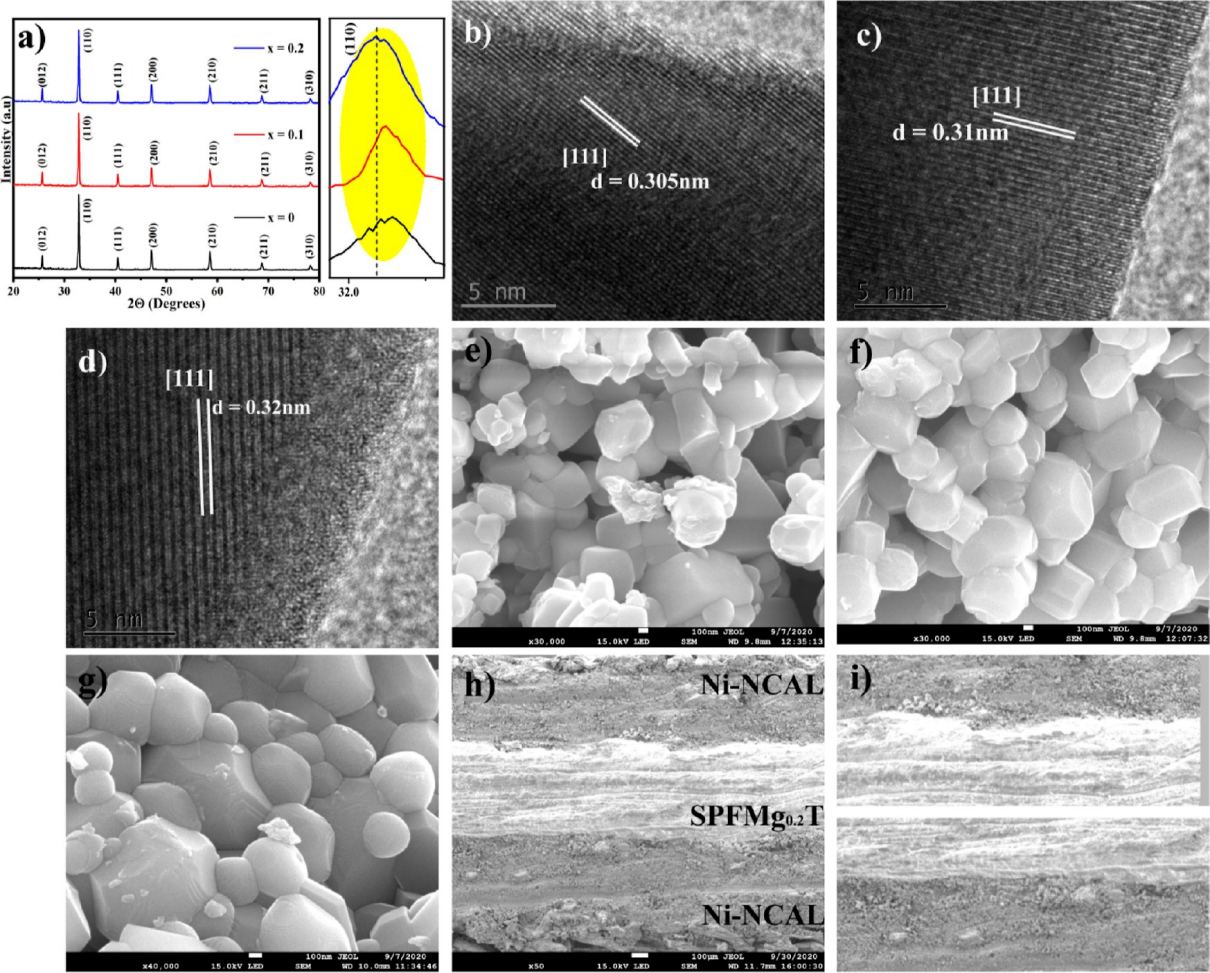
XRD patterns of powders (a); microstructure analysis at the (111) plane obtained by HR-TEM of Sr_0.5_Pr_0.5_Fe_0.4_Ti_0.6_O_3−δ_ (b), Sr_0.5_Pr_0.5_Fe_0.3_Mg_0.1_Ti_0.6_O_3−δ_ (c), and Sr_0.5_Pr_0.5_Fe_0.2_Mg_0.2_Ti_0.6_O_3−δ_ (d), SEM images for powders of Sr_0.5_Pr_0.5_Fe_0.4–*x*_Mg_*x*_Ti_0.6_O_3−δ_ (*x* = 0, 0.1, and 0.2) respectively (e–g); and SEM cross-sectional view and interface formation of the fuel cell device with the structure Ni–NCAL/Sr_0.5_Pr_0.5_Fe_0.2_Mg_0.2_Ti_0.6_O_3−δ_/NCAL–Ni (h,i).

XRD patterns of the as-prepared Sr_0.5_Pr_0.5_Fe_0.4–*x*_Mg_*x*_Ti_0.6_O_3−δ_ (SPFT, *x* = 0, 0.1, and 0.2, i.e., SPFT, SPFMg_0.1_T, and SPFMg_0.2_T, respectively) and the peak at the (110) plane in the small window are shown in Figure 1a. SPFT possesses a cubic perovskite structure with the space group *Pm*3̅*m*. Diffractions corresponds to (012), (110), (111), (200), (210), (211), and (310) planes, respectively.^14,17^ No impurity phase or secondary diffraction was observed in the XRD pattern. Thus, Mg was successfully doped (by substitution) into the crystal lattice of SPFT without altering its crystal structure. Nevertheless, a slight shift toward the lower Bragg angle is observed, which is attributed to the lattice expansion due to the bigger ionic radii of Mg^2+^ (0.72 Å)^30^ than that of Fe (0.65 Å),^31,32^ as shown in a small window of Figure 1a. Moreover, Figure 1a is included in the Supporting Information as Figure S1 to clearly illustrate the small peak shift. Crystal size was calculated using the Debye–Scherrer equation
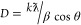
where ƛ is the incident X-ray wavelength, *K* is the constant factor with a value of 0.89, β is the half-width of diffractions, and θ is the most intense diffraction attributed to the (110) plane.

The distribution of particles and grain size was investigated by employing TEM. Figure S2a–c shows representative TEM images of the three as-prepared SPFT samples, displaying the nanoscale particles. SPFT consists of irregularly shaped particles with particle sizes ranging from nano- to micro-scale. In SPFMg_0.1_T, agglomeration of the particles significantly decreased compared to that in SPFT (Figure S2b). In SPFMg_0.2_T, faceted and regular shapes with compacted distribution and contacts can be observed in Figure S2c. The microstructure of SPFT, SPFMg_0.1_T, and SPFMg_0.2_T was further studied via HR-TEM, as depicted in Figure 1b–d. The fringes with lattice spacing are identified and displayed in Figure 1b–d with values of 0.305, 0.31, and 0.32 nm and labeled in the HR-TEM images, corresponding to the (111) planes of SPFM, SPFMg_0.1_T, and SPFMg_0.2_T, respectively.

Overall, the surface morphology and distribution of particles and their size differ for SPFT, SPFMg_0.1_T, and SPFMg_0.2_T (Figure 1e–g). Increasing the Mg dopant concentration makes the surface more uniform and decreases the particle size. This is indeed beneficial for the overall electrochemical performance of the fuel cell. SPFMg_0.2_T showed low agglomeration compared to SPFT and SPFMg_0.1_T, which is possibly due to the purification caused by the self-diffusion process as Mg doping resulted in dispersion from the inside to the surface. As the Mg dopant concentration increases, particle size adjusts, thus restraining the agglomeration. In general, doping is of crucial importance in the amendment of the electronic structure of the respective material; therefore, these parameters can be controlled via the modulation of the doping concentration.^33^ The 20% Mg doping (SPFMg_0.2_T) resulted in the uniformity of the particles and formed coherence and good adhesion, which is ideal for establishing a network and transport pathway for the charge carriers’ transport in the material used as an electrolyte for SOFCs. As reported, small particles possess a large surface active area to form massive contacts between the electrode and electrolyte composite.^34^ Indeed, in SPFMg_0.2_T, the formation of grains is limited. Moreover, the cross-sectional view SEM image of the fuel cell device based on Ni–NCAL/SPFMg_0.2_T/Ni–NCAL is displayed in Figure 1h, where a gas-tight electrolyte layer without obvious cracks is sandwiched between the two porous electrodes. However, the surface of the electrolyte layer is shown to be rough due to the scissoring of the cell for SEM analysis using a blade, which led to a rough surface appearance. In addition, the interfaces of the electrolyte with both electrodes, either at the anode or cathode, demonstrate the formation of fine interfaces, as shown in Figure 1i. More importantly, the particle distribution of the electrolyte layer of SPFMg_0.2_T within the cell is shown in Figure S2d, which clearly illustrates that the electrolyte will stop the crossover of the H_2_–O_2_ gases. Besides, the relative density of sintered pellets of SPFT, SPFMg_0.1_T, and SPFMg_0.2_T was calculated using the lattice volume from XRD and Archimedes’ principle. It showed higher values of the relative density of SPFMg_0.2_T (88%) than that of SPFMg_0.1_T (81%) and SPFT (74%) sintered at LTs, respectively. This gives the evidence that SPFMg_0.2_T possesses suitable gas tightness and is prone to be employed as an electrolyte to avoid the H_2_–O_2_ crossover during the fuel cell operation.

EDS was conducted to determine the elemental distribution by considering their respective high-angle angular dark-field (HAADF) image and their mixed color image, as shown in Figure S3a,b. The corresponding elements such as Sr, Pr, Fe, Mg, and Ti and the presence of oxygen content in the composition are displayed in Figure S3c–h, which roughly confirms the optimal composition of materials.

### 3.2 Chemical States Analysis

**Figure 2:**
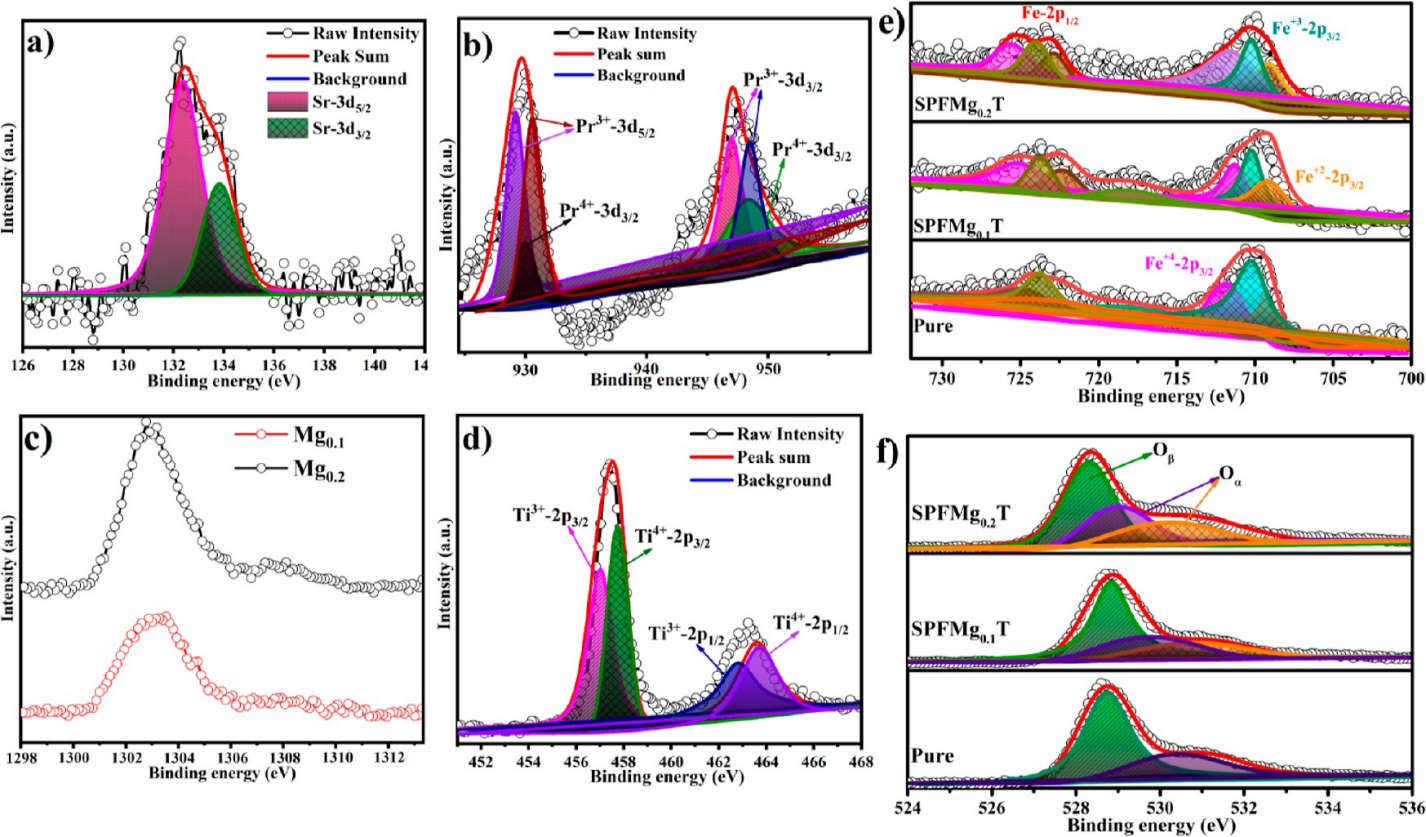
XPS spectra of individual elements: Sr 3d (a); Pr 3d (b); Mg 1s (c); Ti 2p (d); Fe 2p (e); and O 1s (f) of Sr_0.5_Pr_0.5_Fe_0.4–*x*_Mg_*x*_Ti_0.6_O_3−δ_ [*x* = 0, 0.1, and 0.2].

XPS was used to probe the chemical and oxidation states of each element in their respective compositions, where Figure S4 shows the complete survey spectra of SPFT, SPFMg_0.1_T, and SPFMg_0.2_T with characteristic peaks of Sr, Pr, Fe, Mg, Ti, and O, respectively. More detailed characterization was conducted by Gaussian functions and the Shirley background, as shown in Figure 2. Initially, the Sr 3d spectrum can be deconvoluted into two spectra, which correspond to the two characteristic peaks maintaining Sr 3d_3/2_ and Sr 3d_5/2_ at the binding energies (BEs) of 132.5 and 134.4 eV, respectively (Figure 2a). Figure 2b shows the persistence of two Pr 3d distinctive peaks at the surface of respective compositions, such as Pr^4+^ accredited to 3d_5/2_ at 931.6 eV and 3d_3/2_ at 949.2 eV.^35^ In more detail, Pr^3+^ can be ascribed to the Pr 3d_5/2_ peak pairs at 933.3/928.4 eV and Pr 3d_3/2_ peak pairs at 950.1/947.2 eV. This suggests that Pr 3d has 3+ and 4+ oxidation states. Also, the regions of Pr^3+^ peaks are substantially greater than those of Pr^4+^ peaks. As reported, the reduction of higher-valence-state cations to lower-valence-state cations can assist in the creation of oxygen vacancies along with the increase of temperature.^27,36−38^ After Mg doping, the Mg 1s BE peak (detected at 1303.4 eV) appeared and is assigned to Mg^2+^, as shown in Figure 2c. An increase in the intensity and widening of the peak was observed due to the successful doping of Mg in SPFMg_0.1_T and SPFMg_0.2_T. Figure 2d shows the investigated chemical state of Ti^3+^ by XPS measurement. Figure 2d represents the Ti^3+^ state where two prominent peaks of the typical specimen spectra of Ti 2p appeared, that is, peaks situated at 464.3 and 458.5 eV can be assigned to Ti 2p_1/2_ and Ti 2p_3/2_, respectively. In addition, two low-intensity peaks appeared under each prominent peak at 465.4 eV for Ti 2p_1/2_ and 458.8 eV for Ti 2p_3/2,_ corresponding to Ti^4+^. Thus, the presence of different oxidation states of Ti 2p plays a vital role in the enrichment of the oxygen vacancies in the proposed materials.

Figure 2e shows the Fe 2p spectra, where Fe^2+^ 2p_1/2_ and Fe^2+^ 2p_3/2_ are situated at 709.2 and 722 eV, respectively, and Fe^3+^ 2p_1/2_ and Fe^3+^ 2p_3/2_ are situated at 710.963 and 725.1 eV, respectively.^39^ In addition, the Fe^4+^ 2p_1/2_ and Fe^4+^ 2p_3/2_ peaks are also associated with the BEs of 712.96 and 725.31 eV, respectively, with a satellite peak at 717.9 eV.^40^ These findings indicate that Fe 2p exists in three different oxidation states (Fe^2+^, Fe^3+^, and Fe^4+^) at their respective BEs, as shown in Figure 2e, and there are different percentages of Fe 2p oxidation states in each material. Moreover, the Fe 2p peaks in SPFMg_0.2_T are shifted to a lower BE, and a BE downshift of 0.75 ± 0.05 eV was observed in SPFMg_0.2_T compared to that in SPFT. A slight shift was observed with the increment of Mg doping and the average valence state of Fe changing.^41^ As the result, the B-site adjustment of cations via the partial substitution of Fe with Mg leads to the formation of more oxygen vacancies. Moreover, the existence of mixed oxidation states of Fe (Fe^4+^/Fe^3+^ and Fe^3+^/Fe^2+^) play a crucial role in our materials’ overall electrochemical performance.

At last, O 1s spectra are displayed in Figure 2f. After Mg doping, the O 1s spectra of SPFMg_0.1_T and SPFMg_0.2_T are widened and shifted toward a higher BE than that of SPFT. In general, the ionic conduction of the material is significantly affected by the content of oxygen vacancies, as reported previously.^42−44^ For instance, Dong and Barr et al. attributed the BE in the 528–529.5 eV range to the lattice O and highly oxidant O in the materials.^22,43^ Similarly, the lattice oxygen can be represented by peak O_β_ at the BE of 529 eV. While the surface oxide defects or surface oxygen species adsorbed on the oxygen vacancies can be depicted by peak O_α_ at the surface, including oxygen ions in the oxygen vacancy region and the intermediate oxygen oxidation state related to the −OH functional group, O^*x*–^ (0 < *x* < 2), at BEs of 530.2 and 531 eV, respectively.^45^ Therefore, in our case, it can be observed that with the increase of Mg doping, a peak broadening occurred toward higher BE, as shown in Figure 2f, where the surface-active oxygen is also improved, causing the production of oxygen vacancies.^46^ After the calculation, it was observed that the relative ratio value of O_α_ and O_β_ gradually increased from 1.11 to 1.24 for SPFT and SPFMg_0.2_T, respectively. This indicates the increment of chemisorbed oxygen species in SPFMg_0.2_T. Thus, the increment of chemisorbed oxygen vacancies leads to increased ionic conduction in the SPFMg_0.2_T electrolyte, where the chemisorbed oxygen vacancies can easily be liberated during operational temperatures. The exposure of surface oxygen vacancies leads to the transport of oxygen ions in the SPFMg_0.2_T electrolyte. According to the literature, normally, the conduction of oxygen ions in solid oxides takes place in forms of vacancy diffusion,^47^ in which the oxygen vacancies are mostly created/supplied by oxygen defects. In this regard, the oxygen defective material can provide diffusion pathways to generate high ionic conductivity. Therefore, the formation of surface oxygen defects and chemisorbed oxygen species substantially assist in the fast transport of oxygen ion conductions in the as-prepared electrolyte materials.

Moreover, the O 1s spectra of the raw powder and the powder scratched off the SPFMg_0.2_T electrolyte cell after the fuel cell performance being presented in Figure S5a,b. The amount of the oxygen vacancies and −OH functional group are dominant compared to that in pure powder. Therefore, in a fuel cell operation, high oxygen vacancies are responsible for high oxide ion conductivity. Overall, the designed SPFMg_0.2_T electrolyte possesses all the characteristics of an efficient fuel cell device, that is, a high amount of oxygen vacancies that result in high ionic conductivity.

### 3.3 Electrochemical Performance

**Figure 3:**
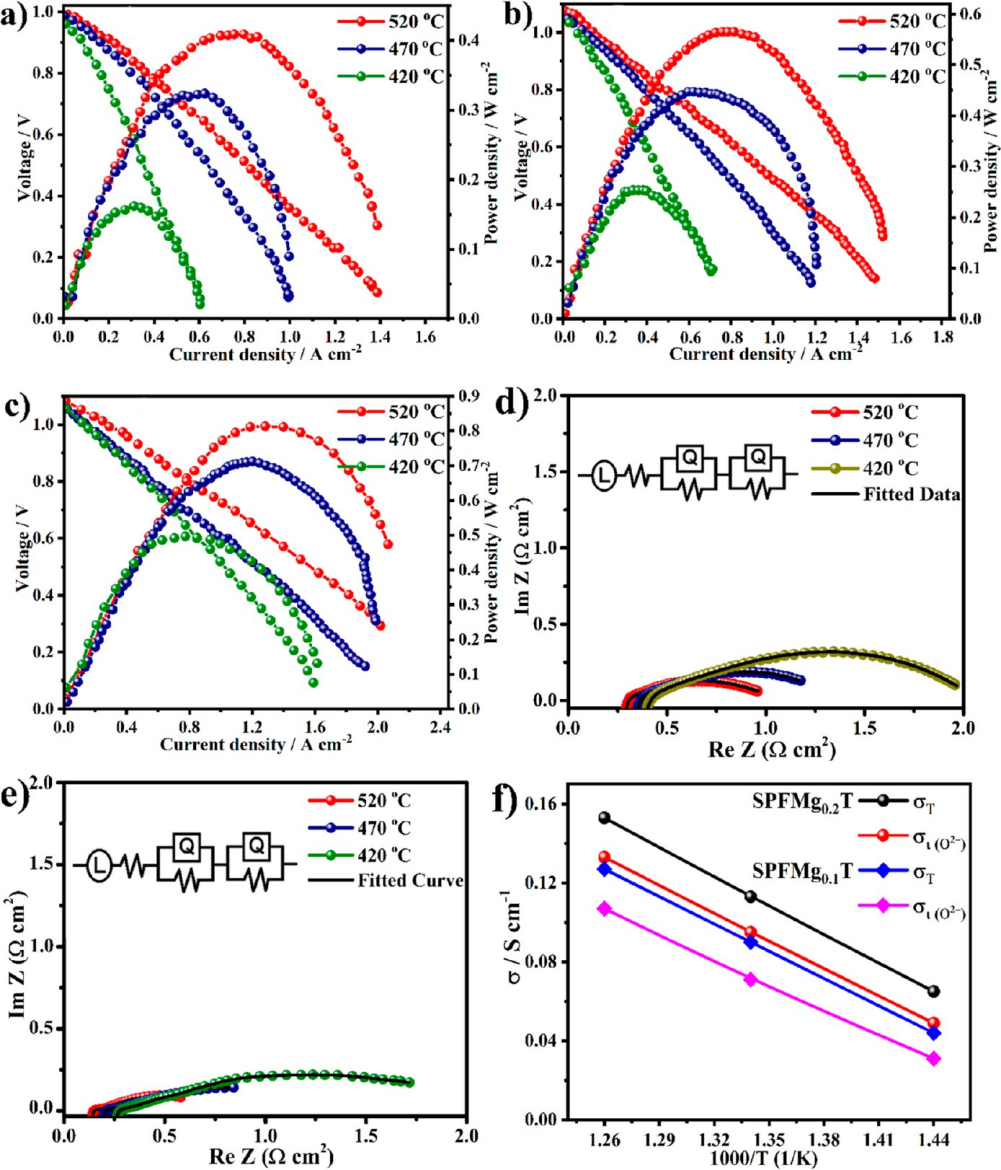
Electrochemical performance in terms of *I*–*V* and *I*–*P* curves for the Sr_0.5_Pr_0.5_Fe_0.4_Ti_0.6_O_3−δ_ cell (a), Sr_0.5_Pr_0.5_Fe_0.3_Mg_0.1_Ti_0.6_O_3−δ_ cell (b), and Sr_0.5_Pr_0.5_Fe_0.2_Mg_0.2_Ti_0.6_O_3−δ_ fuel cell (c) at an operating temperature of 520–420 °C; EIS spectra of Sr_0.5_Pr_0.5_Fe_0.3_Mg_0.1_Ti_0.6_O_3−δ_ (d) and Sr_0.5_Pr_0.5_Fe_0.2_Mg_0.2_Ti_0.6_O_3−δ_ (e) at the temperature of 520–420 °C in the H_2_ and air environment; and total and ionic conductivities of Sr_0.5_Pr_0.5_Fe_0.2_Mg_0.2_Ti_0.6_O_3−δ_ and Sr_0.5_Pr_0.5_Fe_0.3_Mg_0.1_Ti_0.6_O_3−δ_ at 420–520 °C (f).

The designed SPFT, SPFMg_0.1_T, and SPFMg_0.2_T samples were assessed in the SOFC operation to evaluate the feasibility of the prepared electrolyte materials through the doping approach. Figure 3 presents the *I*–*V* characteristics for the fuel cells using SPFT, SPFMg_0.1_T, and SPFMg_0.2_T electrolytes at 420–520 °C along with the fuel cell electrochemical performances after stabilizing the OCVs of the cells. In Figure 3a, the fuel cell based on the SPFT electrolyte yielded a power density of 0.482 W cm^–2^ with an OCV of 1.0 V at 520 °C. An improved power density of 0.698 W cm^–2^ with a higher OCV of 1.043 V at 520 °C was achieved using the SPFMg_0.1_T electrolyte, as shown in Figure 3b. The performance was further enhanced using the SPFMg_0.2_T electrolyte with the power density of 0.83 W cm^–2^ at 520 °C, as shown in Figure 3c. According to our experimental observation, the OCV of the SPFMg_0.2_T fuel cell reached 1.06 V within 37 s after the supply of H_2_/air. Both SPFMg_0.1_T and SPFMg_0.2_T SOFCs showed an OCV of >1.04 V, thus ruling out the possibility of the cell short-circuiting because the semiconductor was used as an electrolyte layer. Nevertheless, the obtained OCV of SPFT decreased to 0.98 V with the lowering of the operating temperature to 420 °C, which is due to the presence of certain electronic characteristics in SPFT. Electronic conduction is known to be detrimental to the fuel cell device, which causes the short-circuiting trouble. However, the measurement found that the introduction of Mg into SPFT can promote the fuel cell OCV, possibly due to the charge compensation mechanism and energy band alignment playing a crucial role in charge transport—discussed later in this work.

More attractively, the enhanced electrochemical power output is ascribed to the Mg doping; where the intermediate concentration of Mg doping (20% of Mg in SPFMg_0.2_T) has displayed better and stable fuel cell performance in terms of power density and OCV as compared to a lower doping concentration (10% of Mg in SPFMg_0.1_T). Furthermore, Figure 3c shows the peak power density of the optimal composition of the SPFMg_0.2_T electrolyte evaluated in the fuel cell, and it can be seen that the power density increases from 0.51 W cm^–2^ at 420 °C to 0.83 W cm^–2^ at 520 °C as a result of the thermally activated ion transportation accompanied by high OCVs maintained at levels above 1.06 V at each testing temperature. However, the power output can also be restricted due to the high thickness of the electrolyte (approx. 500 μm). The fuel cell based on the SPFMg_0.1_T electrolyte demonstrated an inferior performance compared to that of the SPFMg_0.2_T one, which should be mainly due to the low concentration of Mg doping. The possible reason can be the acceptable higher doping ratio that leads to the creation of oxygen vacancies and consequently improves the ionic conduction (this is supported by the XPS data shown in Figure 2). Based on our side experiments (Figure S6a–c), a further increase in the doping concentration to above 20% has substantially decreased the fuel cell electrochemical performance. Briefly, the above analyses indicate that the SPFMg_0.2_T electrolyte membrane offered the highest power output and a stable and higher OCV, manifesting that it can be used as a functional electrolyte in the LT-SOFCs. Besides, the peak power densities of fuel cells show an upward trend with the increase of Mg content in the electrolyte from 0 to 20%, where the further increase of the Mg content (from 20 to 40%) in the materials led to the decrease in the power output of the fuel cell, as presented in Figure S6a–c. Furthermore, EIS was employed to understand the in-depth electrical properties of the utilized materials, as discussed below.

As reported herein, the obtained favorable power output of 0.83 W cm^–2^ for the SPFMg_0.2_T electrolyte in a fuel cell is a competent and functional electrolyte for LT-SOFC uses. Therefore, we further evaluated the prepared SPFMg_0.2_T electrolyte at 520 °C. The fuel cell device based on the SPFMg_0.2_T electrolyte displayed the operation for 140 h at 520 °C under a constant current density of 120 mA cm^–2^ after the fuel cell device’s transient-state operating condition and it is discussed later in detail. By integrating the optimized content of Mg into SPFT, the SPFMg_0.2_T system can be a successful and functional electrolyte in terms of power output in LT-SOFCs.

### 3.4 Electrochemical Impedance Spectroscopy

The EIS spectra were measured from 420 to 520 °C in a H_2_ and air environment in the OCV condition, as shown in Figures S7a and 3d,e. As previously reported, the attained EIS curves define three dominant processes, indicated by intersects at lower frequency (LF), intermediate frequency (IF), and higher frequency (HF) regions, representing the mass transfer resistance (*R*_2_), charge transfer resistance (*R*_1_), and Ohmic resistance (*R*_o_), respectively.^48,49^ The extracted information from raw data via the equivalent circuit LR_o_QR_1_QR_2_ are listed in Tables S1 and S2, where *L* is the inductance, *R*_o_ is the Ohmic resistance, *Q* is the constant-phase element (CPE) representing the non-ideal capacitance, and *R*_p_ = *R*_1_ + *R*_2_ is the polarization resistance involved in charge and mass transfer. The CPE is a capacitive element with a frequency-independent negative phase between current and voltage, which interpolates between a capacitor and a resistor. The capacitance value for each process was obtained using the equation
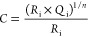


The obtained *C* for (*R*_1_*Q*_1_) and (*R*_2_*Q*_2_) can be related to the charge transfer and electrode polarization process, as reported elsewhere;^50^ according to the obtained *C*_i_, and the semicircles denoted by (*R*_1_*Q*_1_) and (*R*_2_*Q*_2_) can be ascribed to the grain boundary and electrode polarization processes, respectively. Further, “*n*” is the depressed arcs and the calculated capacitance values. However, it can be observed that the Ohmic resistance *R*_o_ of SPFMg_0.2_T and polarization resistances (*R*_p_) are lower than that of SPFMg_0.1_T, enlisted in Tables S1 and S2. Nevertheless, the *R*_o_ and *R*_p_ of the SPFT electrolyte are comparatively higher than that of SPFMg_0.1_T and SPFMg_0.2_T. The above results evidence that SPFMg_0.2_T has lowered Ohmic and polarization resistance compared to that of SPFMg_0.1_T and SPFT, due to the presence of the high amount of oxygen vacancies, which leads to the enhancement of ionic conduction in SPFMg_0.2_T. In addition, the introduction of Mg into SPFT demonstrated a reduction of electrode polarization compared to that of SPFT, proposing the high catalytic activity of the utilized electrodes. Particularly, the lower charge transfer resistance of the SPFMg_0.2_T cell than that of the SPFT cell can be attributed to the replacement of the Fe^3+^ ions by the lower valence Mg^2+^ ions. Therefore, the replacement of Fe^3+^ via Mg^2+^ established a compensation of the oxidation state, leading to the generation of Fe^4+^ (holes) ions (Fe^3+^ → Fe^4+^ + Mg^2+^), which provides an effective path for the carrier concentrations. Besides, the area-specific resistance of Mg-doped SPFT was reduced. Moreover, the well-functioning electrolyte SPFMg_0.2_T has a distinct contribution to the enhancement of catalytic activity, which participates in the electrode reaction and promotes the ORR and HOR activities at the electrolyte/electrode interface regions. Besides, the oxide ion conducting characteristics were also evaluated by analyzing SPFMg_0.2_T as the electrolyte cell in air/air and H_2_/air atmospheres, as shown in Figures S7b and 3e, respectively. It can be seen that initially, the SPFMg_0.2_T-electrolyte-based fuel cell exhibited large Ohmic and polarization resistances, but when the environment was changed from air/air to H_2_/air for the same cell and allowed for 0.5 h, both Ohmic and polarization resistances reduced significantly in the H_2_/air atmosphere as compared to that in the air one. Indeed, the exposure of SPFMg_0.2_T to the H_2_/air atmosphere induced phase transition to an ion-conducting phase. Thus, this provides the evidence that this material possesses high ionic conductivity in the fuel cell environment, which is favorable for the SOFC operation.

In this work, the EIS analysis performed in H_2_/air is used to determine the total conductivity (σ_t_), which is enhanced from 0.06 to 0.153 S cm^–1^ of the SPFMg_0.2_T fuel cell in the temperature range of 420–520 °C, as shown in Figure 3f. In addition, we utilized an unconventional method to calculate the ionic conductivity of SPFMg_0.1_T and SPFMg_0.2_T. Figure 3f shows how the Ohmic resistance was utilized to assess and determine the ionic conductivity value based on the obtained respective *I*–*V* polarization curves. The total Ohmic polarization losses (Δ*V*_ohm_) of the tested cells are replicated by considering the linear section of the polarization curve of the fuel cell curve at the low-intermediate current region, which is mainly caused by the Ohmic resistance of the electrode and electrolyte.^51^ The electronic resistance offered by the electrodes in the form of NCAL/Ni-foam is insignificant compared to the ionic resistance of the presented SPFMg_0.2_T electrolyte. The total Ohmic resistance acquired from the polarization curve is presumably equivalent to the ionic resistance provided by the SPFMg_0.2_T electrolyte. As a result, the area-specific resistance (*R*_ASR_) of the SPFMg_0.2_T electrolyte is calculated via *R*_ASR_ = Δ*V*_ohm_/Δ*I*_ohm_, where the Δ*V*_ohm_ represents the Ohmic polarization losses and Δ*I*_ohm_ corresponds to the current drop,^52^ displayed by the slope of the *I*–*V* characteristics curve in the Ohmic polarization part. From this, the ionic conductivity (σ_i_) of SPFMg_0.1_T and SPFMg_0.2_T electrolytes can be estimated according to the following equation based on the *I*–*V* curves
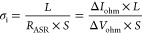
where *L* and *R* are the thickness of the electrolyte layer and resistance, respectively. *S* denotes the active area of the pellet. The electrical conductivities display the ionic conductivities of SPFMg_0.1_T and SPFMg_0.2_T and were determined from the respective *I*–*V* curves shown in Figure 3b,c. Afterward, the calculated ionic conductivities of SPFMg_0.1_T and SPFMg_0.2_T are 0.107–0.031 and 0.133–0.049 S cm^–1^ at 520–420 °C, respectively, as presented in Figure 3f. The obtained ionic conductivity of SPFMg_0.2_T is remarkable and higher than that of the latest reported simplex and composite electrolytes, such as SFT–SDC (0.1 S cm^–1^ at 520 °C), GDC and YSZ (5.8 × 10^–3^ and 1.1 × 10^–3^ S cm^–1^ at 500 °C, respectively), and SiC–ZnO (0.12 S cm^–1^ at 550 °C).^14,53,54^ The high ionic conduction is associated with the doping of Mg ions into SPFT, which leads to the charge compensation mechanism, resulting in the creation of higher oxygen vacancies. The XPS analysis also gives a hint for the enhancement of oxygen vacancies. Similarly, the high ionic conduction trend was observed with doping of Mg into LiCoO_2−δ_ and doped ceria;^55^ therefore, a similar mechanism is followed here. The obtained ionic conductivity disclosed the ability of Mg doping into SPFT as an electrolyte. Indeed, the enhancements of ionic conduction and avoiding the short-circuiting issue of the as-prepared materials have an enormous contribution to energy band engineering. Therefore, we discuss the impact of designing single-phase perovskite semiconductor materials based on the energy band structure in detail.

### 3.5 Synergistic Effect of Mg Doping

**Figure 4:**
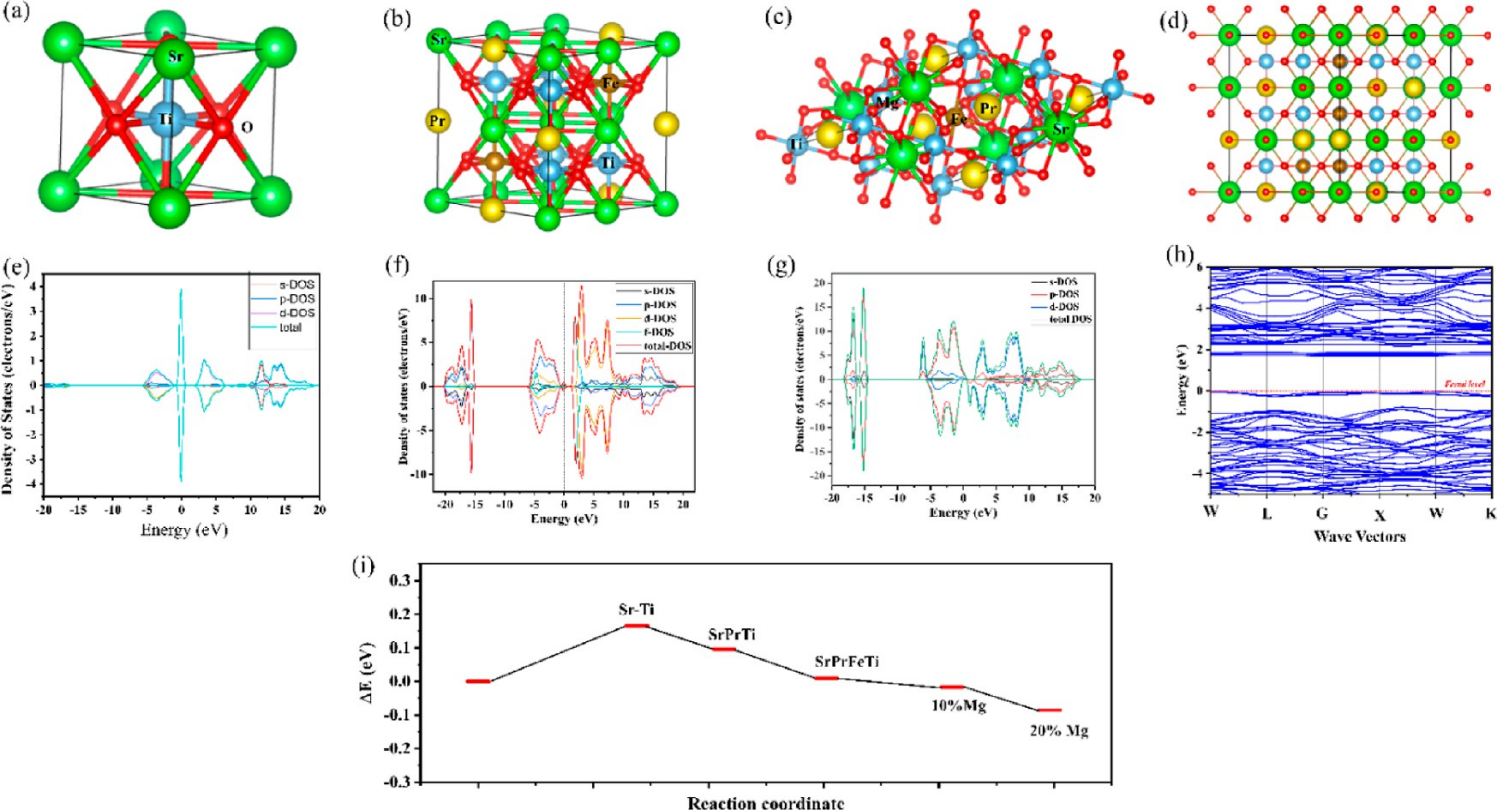
Optimized structure of SrTiO_3_ (a); Sr_0.5_Pr_0.5_Fe_0.4_Ti_0.6_O_3_ (b); 10% Mg-doped Sr_0.5_Pr_0.5_Fe_0.4_Ti_0.6_O_3_ (c); and 20% Mg-doped Sr_0.5_Pr_0.5_Fe_0.4_Ti_0.6_O_3_ (d) structures, respectively. Calculated partial DOS (PDOSs) for different orbitals for the optimized structures of Sr_0.5_Pr_0.5_Fe_0.4_Ti_0.6_O_3_, 10% Mg-doped Sr_0.5_Pr_0.5_Fe_0.4_Ti_0.6_O_3_, and 20% Mg-doped Sr_0.5_Pr_0.5_Fe_0.4_Ti_0.6_O_3_ structures (e–g), respectively. Energy band structure of 20% Mg-doped Sr_0.5_Pr_0.5_Fe_0.4_Ti_0.6_O_3_ structures by DFT calculations (h). Calculated relative diffusion energy for the oxygen ions at different sites and structures such as SrTiO_3_ (Sr–O–Ti), Sr_0.5_Pr_0.5_TiO_3_ (Pr–O–Ti), Sr_0.5_Pr_0.5_Fe_0.4_Ti_0.6_O_3_ (Fe–O–Ti), 10% Mg-doped Sr_0.5_Pr_0.5_Fe_0.4_Ti_0.6_O_3_ (Fe–O–Mg), and 20% Mg-doped Sr_0.5_Pr_0.5_Fe_0.4_Ti_0.6_O_3_ (Fe–O–Mg) structures, respectively (i).

To gain an in-depth insights into the electron distribution state utilizing Mg doping, the DOS for each orbital is calculated by the DFT to investigate the responsible reasons for the enhanced high ionic conductivity. Figure 4a–d displays the optimized structures of SrTiO_3_, SPFT, SPFMg_0.1_T, and SPFMg_0.2_T, respectively. After Mg doping, the crystal structures for SPFT (Figure 4b–d) are similar to that of pristine SrTiO_3_ with lattice distortion. Meanwhile, the representative DOS values for pure SPFT (b), SPFMg_0.1_T (c), and SPFMg_0.2_T (d) structures were calculated, and it is observed that the DOS value gradually increased near the Fermi level. This corresponds to enhanced chemical activity for the absorption of oxygen ions, as depicted in Figure 4e–g. Moreover, the DOS plots of SPFT reveal that Fe and Mg doping could narrow band gaps between the valence and conduction bands, corresponding to the gradually increased electron concentration. Therefore, the electronic structure of SPFMg_0.2_T was mainly analyzed (Figure 4h), which shows an energy gap of approx. 1.69 eV. Long-distance oxygen vacancies in typical structures, such as SrTiO_3_, prevent simultaneous occupancy of two O_2_ sites. As a result, distinct O sites partially occupy O_2_^2–^ and O_1_^2–^, resulting in heterogeneous coordination of the d-metal atoms (Fe and Ti). The formation of a random distribution of dopants on octahedral sites in the average crystal structure is indicated by the arrangement of oxygen vacancies on two average oxygen sites. However, as shown in Figure 4i, the vicinity and availability of the two oxygen sites suggest a significant structural disorder of the oxide ions. The various configurations will be randomly distributed along with the structural layers, thus creating complex disordered arrangements.

Therefore, oxygen vacancies with less diffusion energy would help enhance oxygen ion and proton transportation and mobility, which are already discussed in detail with the XPS analysis, as shown in Figure 2e,f. It can be observed that there is a change in the oxidation state of Fe 2p and O 1s spectra with the Mg doping into SPFT. The analysis of the change in oxidation states of Fe 2p involving the mixed oxidation states Fe (Fe^4+^/Fe^3+^ and Fe^3+^/Fe^2+^) that tends toward a lower valence state resulted in oxygen vacancies (depicted in Figure 2e). Furthermore, the O 1s spectra extending toward a higher BE demonstrated the higher content of surface oxygen species along with the O–H group, specifically in SPFMg_0.2_T compared to that in SPFT and SPFMg_0.1_T. Moreover, the comparison of O 1s spectra of SPFMg_0.2_T of pure powder and the powder of SPFMg_0.2_T (scratched from the cell after fuel cell operation) illustrated the formation of high oxygen vacancies in the fuel cell condition comparatively (depicted in Figure S5).

**Figure 5:**
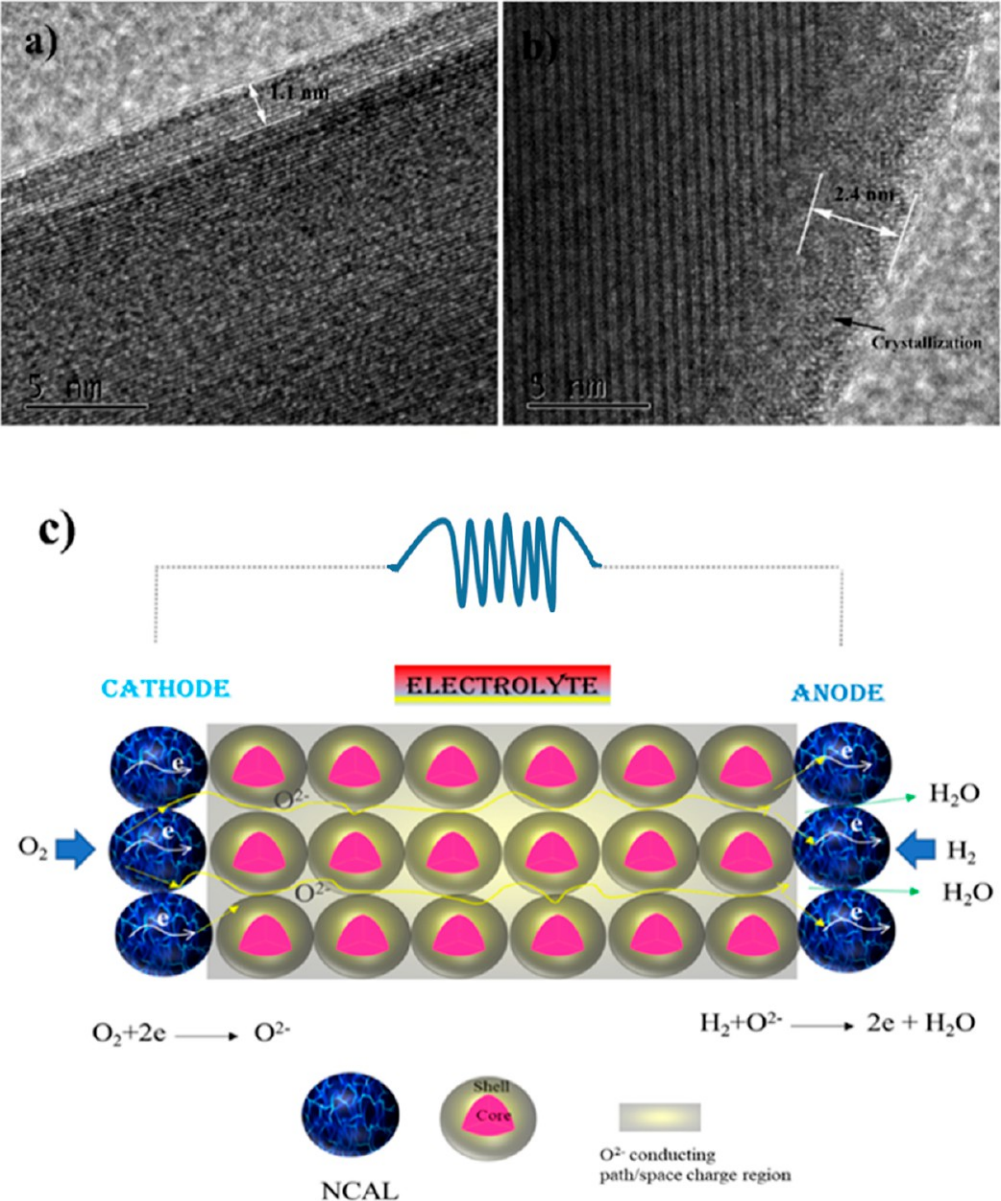
HR-TEM images before and after the fuel cell performance of the SPFMg_0.2_T electrolyte for raw powder (a) and powder scraped from the cell tested in the fuel cell environment (b). Schematic illustration of the electrochemical mechanism of the process occurred at the electrodes and the oxide ion conduction mechanism in the designed cell architecture with the SPFMg_0.2_T electrolyte (c).

As the XPS analysis is one of the sensitive characterization tools, it is used to analyze the surface properties of the materials with the formation of oxygen vacancies, as shown in Figures 2 and S5. In addition, the HR-TEM results of the SPFMg_0.2_T material as-prepared and after the fuel cell performance are depicted in Figure 5a,b. The HR-TEM images suggest that there is a possibility to form oxygen vacancies in SPFMg_0.2_T and the oxygen vacancies are primarily present in the amorphous layer formed at the surface of SPFMg_0.2_T after the fuel cell performance. Figure 5a shows a rough sketch of the surface layer formed, but after the fuel cell performance, there is a clear amorphous layer formed (depicted in Figure 5b), as discussed later via EELS results. Chen et al. reported the formation of the amorphous layer at the La_0.25_Sr_0.75_TiO_3_ powder with a high content of oxygen vacancies and physically characterized it as the core–shell structure.^21^ Similarly, after the fuel cell performance, a wide and clear amorphous layer formed with a high content of oxygen vacancies at the surface of inner bulk SPFMg_0.2_T led to the core–shell structure and behaved with heterostructure characteristics. The XPS (Figures 2 and S5) and HR-TEM (Figure 5) analyses determined a possible high concentration of surface oxygen vacancies created due to the amorphous layer formed at the core of SPFMg_0.2_T powder. In addition, it can be observed in EIS results (depicted in Figures 3e and S7b) that the cell resistances decrease significantly upon the fuel cell environment operation; besides, the surface oxygen vacancies are increased significantly in SPFMg_0.2_T after the fuel cell operation, as depicted in Figure S5b. As this effect can also be understood as a large reduction in the designed cell resistance by changing the air environment to the H_2_/air environment; see Figures 3e and S7b.

Considering the in situ core–shell structure formation of SPFMg_0.2_T, the higher connection among particles or surfaces leads to the creation of a higher number of pathways for the oxide ion conduction, as depicted in the schematic illustration in Figure 5c. Recently, the important results have been reported with a significantly higher ionic conductivity via the ionic conduction by the formation of a heterostructure of CeO_2_/CeO_2−δ_ considering the core–shell structure.^56^ Likewise, there is an ionic conductivity enhancement of oxide ion conduction by forming a heterostructure composite of the ionic conductor and a semiconductor.^57,58^ Thus, enhancing the ionic conductivity in the core–shell structure based on CeO_2_/CeO_2−δ_ is a prominent example of this effect.^56^ Similarly, the same concept of the core–shell structure is being found in SPFMg_0.2_T considering the heterostructure characteristics maintained on the bulk SPFMg_0.2_T and the oxide ion conduction in the amorphous surface layer, filled by the large content of oxygen vacancy defects. This caused the remarkable enhancement of oxide ion conductivity in the optimized SPFMg_0.2_T. There are several concrete shreds of evidence establishing and indicating that the two-dimensional transition metal oxides at the interfaces are entirely different from the interfaces of the typical and conventional semiconductors,^59^ and also, the superconduction phenomenon at the interface has gained significant attention.^60^ In this regard, the state-of-the-art LaAlO_3_/SrTiO_3_ (LAO/STO) heterostructure was reported to illustrate the quasi-two-dimensional (2D) superconduction, where the electronic reconstruction in the 2D LAO/STO interface employed the concept of a “polar catastrophe”.^61,62^ Similarly, the current condition of SPFMg_0.2_T particles involves the core SPFMg_0.2_T covered by an amorphous layer of SPFMg_0.2_T of approx. 2.4 nm thickness. Thus, the core–shell structure formation, where there is a correlation existing between the electron ions in terms of oxygen vacancies can be referred to as the new scientific phenomenon of “ionotronics”. This revealed the new mechanism of electron ion coupling of the materials involving the modification of the structure and properties, which can be achieved via the external fields.^63^ The formation of oxygen vacancies induces the shift in the conduction band for the reduction of energies, as also depicted in the below discussion of energy band alignment. As Mg doping also participates substantially, which plays a crucial role in the development of new physical properties, such as the superconductivity in SPFMg_0.2_T due to the formation of ion defects leading to the change of the electron state and creating new energy levels in the energy band structure.^64^ Moreover, the fuel cell environment (H_2_/air) reduces the energy required for the oxygen vacancy formation in the amorphous shell of the SPFMg_0.2_T electrolyte.^65^ However, such a physical phenomenon of ion conduction is illustrated in Figure 5c. Still, this mechanism of the correlated energy band structure and controlled oxygen defect formation requires more research and investigation in future research work.

**Figure 6:**
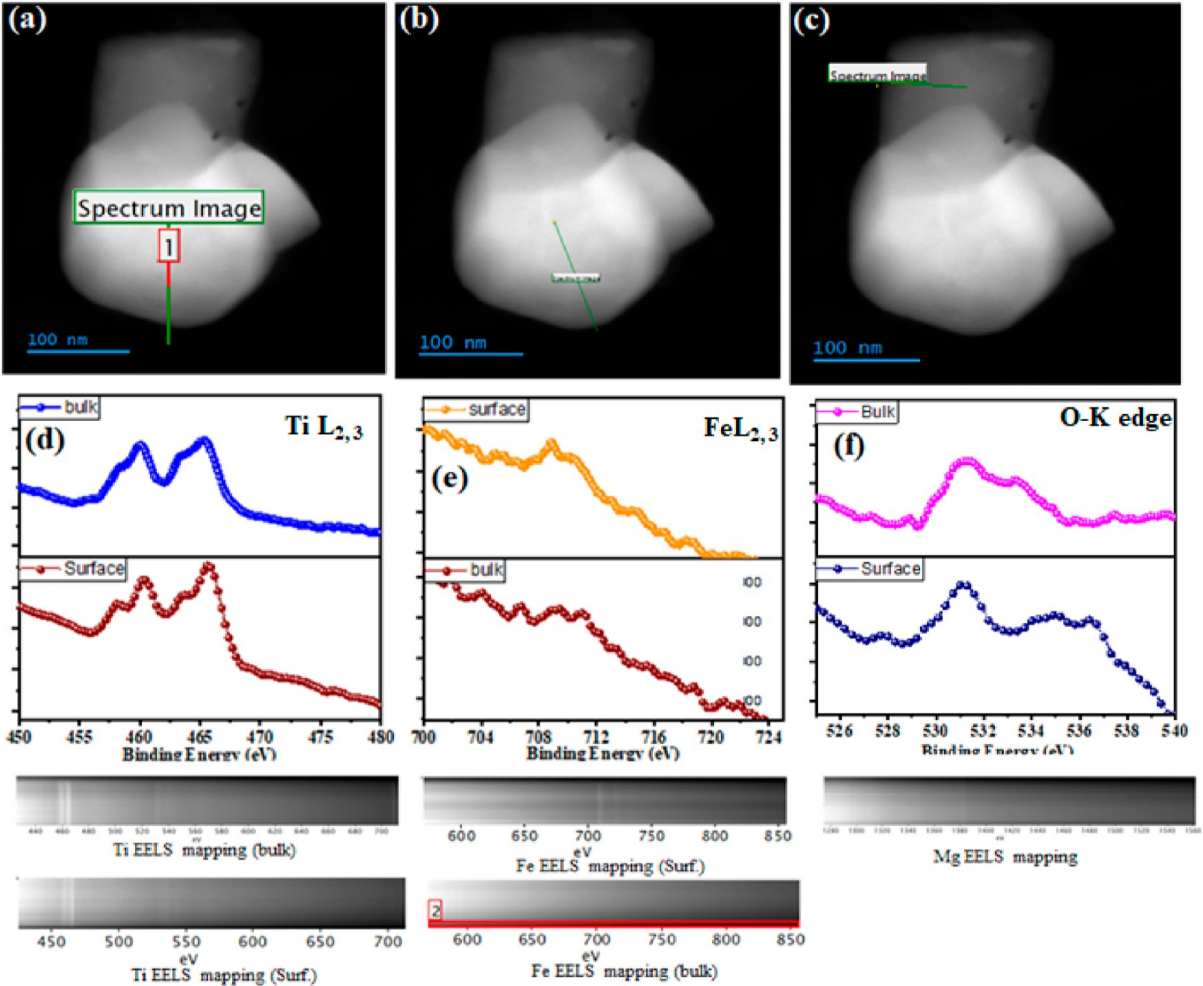
HAADF energy image of the SPFMg_0.2_T electrolyte after the fuel cell performance (a–c); comparison of the Ti L_2,3_-edge, FeL_2,3_-edge, and O K-edge EELS spectra and their corresponding mapping between the grain boundary and bulk of SPFMg_0.2_T (d–f). Adapted from (a–c).

Since the majority of the morphology of the image exhibited a step-like morphology and a high ion-conducting local layer at the surface of the SPFMT electrolyte was formed after fuel cell testing, as presented in Figure 6a–c, it is suggested that there was dominated ionic conduction by the grain boundary due to specific changes in the chemistry or structure, generally an amorphous phase. However, such a phase is occasionally observed. However, EELS data were measured for the SPFMg_0.2_T electrolyte after fuel cell testing. The bulk EELS data for Ti-L_2,3_ suggest that Ti is present in Ti^4+^, while surface EELS data show a mixed valence state of Ti^3+^ and Ti^4+^, as demonstrated in Figure 6d. However, for Fe-L_2,3_, EELS data for the bulk and surface are very different, as shown in Figure 6e. However, it was found that most Fe diffused to the surface layer at grain boundaries, suggesting that the transition metals show changes in the valence as both when approaching the grain boundaries and in the bulk, a feeble signal is observed, as shown in Figure S9. The main structure width of O–K EELS, including the three broad peaks, is evaluated as represented in Figure 6f. As the distance between the bulk to the surface, the positions of the broken lines of the O–K edge correspond to the minimum and maximum in the second differentiated spectra. It seems very reasonable that this shift has also been seen in EELS spectra of other transition metals after reduction in H_2_. By comparing the O–K EELS edge positions at the bulk and surface, we can estimate that the energy shift is proportional to the vacancy concentration. The oxygen vacancy formation in this system played a crucial role in the performance enhancement of the fuel cell device.

### 3.6 Energy Band Structure Mechanism

**Figure 7:**
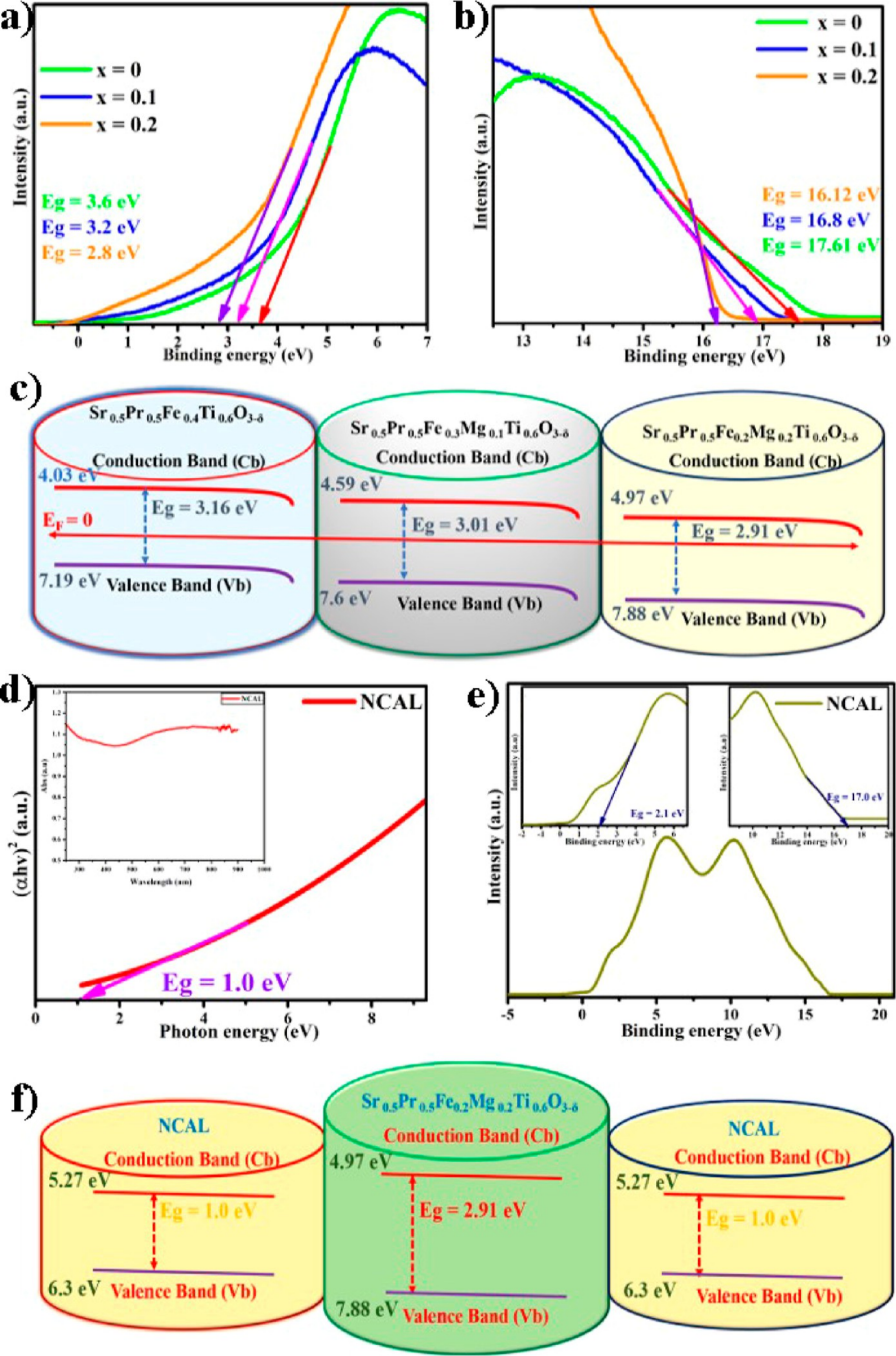
UPS analysis of high- and low-energy intercepts of Sr_0.5_Pr_0.5_F_0.4–*x*_Mg_*x*_Ti_0.6_O_3−δ_ (*x* = 0, 0.1–0.2) (a,b); energy band structure of Sr_0.5_Pr_0.5_F_0.4–*x*_Mg_*x*_Ti_0.6_O_3−δ_ (*x* = 0, 0.1–0.2) (c); UV–visible absorption spectra and band gap view and the high- and low-energy intercepts from the UPS of NCAL (d,e); and energy band structure of the fuel cell device with the structure of NCAL/Sr_0.5_Pr_0.5_F_0.2_Mg_0.2_Ti_0.6_O_3−δ_/NCAL (f).

This work provides a detailed analysis of employing the energy band structure concept to understand and illustrate the mechanism in terms of energy band alignment for the formation of oxygen vacancies that assist in the enhancement of ionic conduction and the stoppage of electronic conduction by altering the valence band maxima via doping of Mg. For this purpose, UV–vis spectroscopy and UPS were employed to understand the energy band structure of the as-prepared materials. Generally, UV–vis spectroscopy was used to obtain the energy band gaps using the following Tauc plots from the optical absorption spectra as below
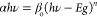
where α, *h*ν, and β_o_ are the absorption coefficient, energy of photons, and energy independent constant, respectively.^66^ The direct or indirect band gap nature of the material can be predicted from the Tauc plot, that is, by plotting (α*h*υ)^*n*^ versus the incident photon’s energy (eV); where “α” is the absorption coefficient, “*h*” is the Planck’s constant, and “υ” is the frequency of the incident photon. Additionally, the superscript is adjusted based on the nature of the band gap, that is, *n* = 2 is used for a direct band gap, while *n* = 1/2 is used for an indirect band gap semiconductor.^67,68^ Since in our case, the prepared material is an indirect band gap semiconductor, we used *n* = 1/2 in the Tauc plot, as shown in Figure S10. The spectra show a negligible lower-energy absorption tail, indicating the material’s indirect band gap nature.^68^ It has been observed in the spectra that the absorption edge for Sr_0.5_Pr_0.5_F_0.4_Ti_0.6_O_3−δ_ is around 440 nm. However, the absorption edge moves toward a higher wavelength with the doping of Mg ions. Figure S10a–c shows the Tauc plots, where the obtained energy band gap values are 2.91, 3.01, and 3.16 eV for SPFMg_0.2_T, SPFMg_0.1_T, and SPFT, respectively. It can be noticed that the energy band gap decreases with the doping of Mg ions, which proposes the creation of intermediate energy levels between the conduction and valence bands. Therefore, in more detail, we utilized UPS to determine valence band maxima, as shown in Figure 7a,b. The valence band (*V*_b_) is calculated using the following equation.^69^
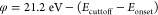
where the *E*_cutoff_ and *E*_onset_ are the high and low BEs of the UPS raw data, respectively. The calculated *V*_b_ values are 7.19, 7.6, and 7.88 eV for SPFT, SPFMg_0.1_T, and SPFMg_0.2_T, respectively. Furthermore, the conduction band minima (*C*_b_) were calculated based on *V*_b_ and the energy band gap. The calculated *C*_b_ values are 4.03, 4.59, and 4.97 eV for the three materials. Thus, all these data presented indicate that the band gap reduction with the appropriate Mg ions may provide an intelligent pathway for charge species. The calculated *V*_b_ and the energy band gaps facilitate constructing the energy band structure (Figure 7c) for different contents of Mg-doped compositions, which depicts that the conduction band narrowed where more oxygen vacancies can be possibly created. Thus, the position of the Fermi level changed; as a result, the valence band shift occurs with Mg doping, and the change in the Fermi level measured from the valence band maximum (EF) and energy of the valence band maximum (EVBM) could enhance the DOS near the Fermi level, as illustrated in the DFT calculations. The easy transport of the charged species from the valence band to the conduction band where more oxygen vacancies are created promotes the fast transport of ions. The band gap reduction is also ascribed to the lowering of the activation energy for the transport of oxide ions.

The above discussion of the energy band structure portrays that the designed energy band structure is feasible for the fuel cell device. For a more in-depth analysis, we applied the energy band theory to understand and describe the constructed fuel cell device that follows the rules of the energy band theory. The fuel cell study revealed that there is a production of extrinsic electrons at the anode side due to the supply of H_2_ fuel; afterward, the produced electrons must flow through the external circuit for the generation of electrical energy. In this regard, certain essential thumb rules must be followed for the successful operation of the fuel cell device to generate high OCV and electrochemical performance. From the context of the energy band theory, there are various cases where electrolytes block electrons, including when the *C*_b_ of YSZ is higher than the redox potential, preventing electron transit.^70^ In this regard, Dong et al. reported a semiconductor TiO_2_ materials to maintain a wide band gap and achieved high electrochemical performance and OCV following the four rules of thumb, such as Cb_anode_ < H_2_/H^+^, Cb_electrolyte_ > H_2_/H^+^, Cb_cathode_ > O_2_/O^2–^, and *V*_b(electrolyte)_ < O_2_/O^2−^.^22,71^ We have carefully designed a new wide-band-gap Mg-doped Sr_0.5_Pr_0.5_Fe_0.2_Mg_0.2_Ti_0.6_O_3−δ_ (SPFMg_0.2_T) sample in light of these four rules, which has significantly followed the energy band theory.

In general, utilizing the energy band theory aims to illustrate that the produced electrons at the anode cannot jump into the electrolyte and follow the external circuit to produce the electrical energy. It is possible due to the high conduction band of the electrolyte as compared to that of an electrode, which avoids any short-circuiting trouble. The lower level of the conduction band can cause e a short-circuiting problem, as observed in SDC.^72^ The band gap (2.91 and 1.0 eV) and valence band (*V*_b_) (7.88 and 6.3 eV) for the SPFMg_0.2_T electrolyte and NCAL electrode were calculated by employing UPS and UV–vis spectroscopy, as shown in Figures 7d,e and S10. The *C*_b_ values (4.97 and 5.27 eV) of the SPFMg_0.2_T electrolyte and NCAL electrode were calculated via the combined *V*_b_ and energy band gaps. It is clearly illustrated that the designed Mg-doped SPFMg_0.2_T electrolyte and NCAL electrode followed the thumb rules of the energy band theory, and the energy band structure (displayed in Figure 7f) was successfully constructed to avoid any short-circuiting trouble and the high electrochemical performance was obtained along with a high OCV.

**Figure 8:**
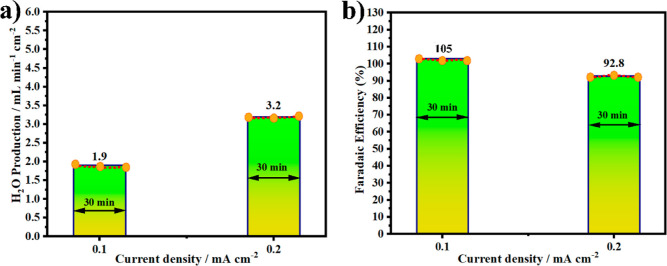
Performance of single cell based on the comparison of H_2_O production through NCAL/SPFMg_0.2_T/NCAL (a). Faradaic efficiency of the single cell H_2_ conversion to H_2_O (b) under different current densities at 520 °C. The average value of three measurements were considered for Faraday efficiency calculation, and three average values are used for the plot.

Furthermore, the individual cell including SPFMg_0.2_T electrolyte with NCAL electrodes demonstrated a high efficiency of fuel conversion under two different current densities. The outlet products were analyzed by on-line GC in 30 min intervals (a GC run repeated every 10 min for three measurements) at each current density. The average value of every three runs was taken as the H_2_O concentration with the 30 min interval for each current density. It can be seen in Figure 8a that the H_2_O concentration production increases with the increase of applied current densities. Moreover, the Faradaic efficiency at a lower current density 0.1 mA/cm^2^ is around 100% but marginally crosses over 100% due to the less production of water resulted of low applied current density, where the slight cross over 100% is also linked to the sensitiveness of the GC–MS instrument(depicted in Figure 8a). Thus, the very low H_2_O concentration produced can easily cause a possible deviation during the calibration and measurement of the GC–MS instrument. However, the increase of the applied current density to 0.2 mA/cm^2^ leads to the increase in the H_2_O concentration production and the obtained Faradaic efficiency of ∼92.8% at 0.2 mA/cm^2^ is still considered high, as shown in Figure 8b.

### 3.7 Interconnection of Electrochemical Performance and Durability

**Figure 9:**
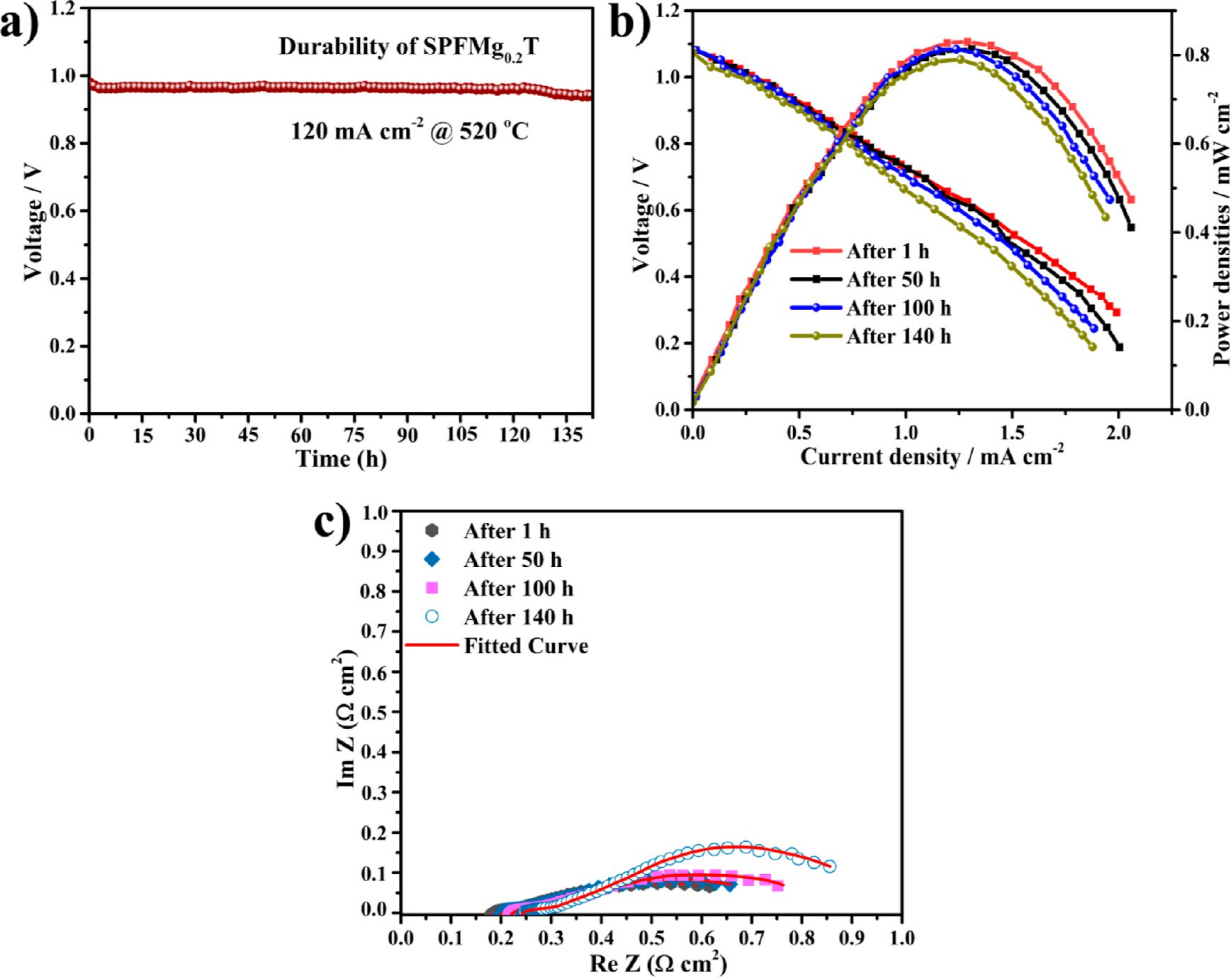
Durability operation of the fuel cell device under a current density of 120 mA cm^–2^ (a) and reproducibility demonstration of fuel cell performance and EIS spectra of the device at different time intervals at 520 °C (b,c) based on the SPFMg_0.2_T electrolyte.

The more essential part of the LT-SOFC is durability, but it is also more complex for practical application, especially in the semiconductor-based fuel cell at the low operational temperature in this emerging energy conversion technology. Therefore, to assess the durable operation of the fuel cell device based on the SPFMg_0.2_T electrolyte with the architecture of Ni–NCAL/SPFMg_0.2_T/NCAL–Ni, the assembled cell is operated at the working temperature of 520 °C under the constant current density of 120 mA cm^–2^. The fuel cell durability has been demonstrated against the time function in the environment of H_2_/air under the optimized flow rate of H_2_ of 110 mL min^–1^, as shown in Figure 9a. It can be observed that the 143 h operation under a constant current density of 120 mA cm^–2^ with a degradation rate of 0.15 mV exhibited a stable operation. In this stability operation, the steady-state operation is considered, and a stable operation of the fuel cell device at 520 °C is observed. It can be regarded that the prominent role of the Mg doping in altering the insulating behavior into the strongly oxide ionic conductor as well as the formation of the core–shell structure play a vital role in the stable and immense support of the stable operation at the obtained working voltage of 0.96 V. However, after the ∼125 h operation, a slight decrease of voltage drop was observed. Still, it is strongly believed that the SPFMg_0.2_T electrolyte-based fuel cell operated at 520 °C can be extended to long-term stable operation. In this regard, several potential technical barriers might lead to the reduced working voltage, resulting in the less stable operation of the fuel cell device: (i) mismatch of the thermal expansion coefficient between the used NCAL electrodes and the SPFMg_0.2_T electrolyte; (ii) possible generation of rusting on the inner surface of the steel chamber of the sample fixture, which may result in increased resistance; and (iii) the difference in the cell geometry due to the lack of engineering facilities to guarantee uniformity of the NCAL–Ni electrode preparation and fuel cell assembly. These all-technical barriers lead to the challenge of reducing cell voltage over a period of time.

Interestingly, we have also performed the fuel cell performance in terms of power output as well as EIS analysis at various time intervals under a long-term operation at 520 °C, but we found that there was a negligible change in the fuel cell power output, OCV, and the Ohmic resistance and polarization resistance, as shown in Figure 9b,c. The prepared fuel cell presented the peak power density of 0.83 W cm^–2^ at 520 °C with the Ohmic resistance of 0.16 Ω cm^2^ and the polarization resistance of 0.43 Ω cm^2^. Afterward, a slight degradation of fuel cell performance was noticed along with Ohmic and polarization resistances, respectively, after 50 h, where the power output was decreased to 0.81 W cm^–2^ without a decline of the OCV and the Ohmic and polarization resistances were 0.18 and 0.46 Ω cm^2^, respectively. After 100 h, the power output was decreased to 0.79 W cm^–2^ and the Ohmic and polarization resistances increased to 0.21 and 0.55 Ω cm^2^, respectively. Subsequently, at 140 h, the power output decreased to 0.77 W cm^–2^ with the decrease of OCV to 1.04 V. In addition, the Ohmic and polarization resistances also increased to 0.25 and 0.63 Ω cm^2^, respectively, which may have happened due to several reasons, but a possible reason could be the fact that in the operating environment, the NCAL electrode on the anode side is liable to be reduced by hydrogen, as our previous reports disclosed, namely, the Ni^2+^ (Co^3+^) easily turned into metallic Ni (Co) through a reduction reaction and was accompanied by the production of active oxygen ions, which reacted with the product H_2_O vapors in the fuel cell reaction to form OH^–^ and then combined with Li^+^ to generate LiOH. The mechanism is similar to the phenomenon that lithium oxide is easily combined with H_2_O vapors to produce LiOH, when the material is exposed to a humidified environment. However, maybe this LiOH results in an insulating phase at the anode and electrolyte interface, which can create an insulating phase to increase the polarization resistance of the fuel cell. However, there is still a need to design new electrodes to overcome the resistances produced, which restrained the fuel cell efficiency. Considering the electrochemical power output, it can be concluded that the employed SPFMg_0.2_T electrolyte-based fuel cell was stable with relatively low resistances. The EIS analysis at different intervals of time of the cell-based on electrolyte SPFMg_0.2_T revealed no severe increase in Ohmic resistances and mass and charge transport resistances, which guarantees better fuel cell performance of the designed electrolyte for the low-temperature based FC device with the durable operation.

## 4 Conclusions

In summary, a new electrolyte Mg-doped Sr_0.5_Pr_0.5_Fe_0.2_Ti_0.6_O_3−δ_ was proposed for LT-SOFCs. The material properties including the phase structure, morphology, microstructure, and surface oxygen vacancies were investigated through XRD, SEM, HR-TEM, and XPS. The ionic conductivity and electrochemical performance of the material were also characterized, which revealed that by using Mg doping, Sr_0.5_Pr_0.5_F_0.4_Ti_0.6_O_3−δ_ could exhibit an improved ionic conductivity and appreciable fuel cell performance with high OCVs. The presented core–shell structure can be considered as a semiconductor core and a surface layer for superionic conduction, portraying the heterostructure characteristics. In addition, the correlation of electron–oxide ions (oxygen vacancies) in the formed core–shell structure–based heterostructure opened a new scientific mechanism for the superionic conduction in the SPFMg_0.2_T electrolyte toward the LT-SOFC, where the interface/surface played a vital role. In addition, the band gap engineering in the design of the energy band structure via Mg doping also had a substantial role in charge separation and ionic transport/transfer. Considering all these possible charge mechanisms has provided efficient evidence of surficial/boundaries-based superionic conduction with an ionic conductivity of 0.133 S cm^–1^ and suppression of electronic conduction that resulted in a significant fuel cell performance of 0.83 W cm^–2^ at 520 °C. Moreover, the GC characterizations-based calculated Faradaic efficiency demonstrated that SPFMg_0.2_T is an efficient electrolyte during the fuel cell operation mode. Thus, the work introduces a new approach for the development of functional electrolytes for LT-SOFCs and helps leverage the consistent advantage of the low cost and facile way for commercialization.

## Supporting Information Available

XRD analysis, TEM and SEM analyses, EDS mapping, XPS analysis, fuel cell performance, EIS analysis, EELS results, optical characteristics, Tables including EIS fitted data of the SPFT electrolyte obtained from Z-Simpwin software at temperatures ranging from 520 to 420 °C, and EIS fitted data of the Sr_0.5_Pr_0.5_Fe_0.4–*x*_Mg_*x*_Ti_0.6_O_3−δ_ (*x* = 0, 0.1, and 0.2) electrolyte obtained from Z-Simpwin software at temperatures ranging from 520 to 420 °C (PDF)

## Notes

The data can be provided upon request.
