# Supplementary material for: Modulating the Energy Band Structure of the Mg-Doped Sr0.5Pr0.5Fe0.2Mg0.2Ti0.6O3−δ Electrolyte with Boosted Ionic Conductivity and Electrochemical Performance for Solid Oxide Fuel Cells
Source: ACS Appl Mater Interfaces. 2022 Sep 19;14(38):43067–84. doi: 10.1021/acsami.2c06565 (PMC9523621; doi:10.1021/acsami.2c06565)
Supplement: Supplementary file 1 — am2c06565_si_001.pdf [file am2c06565_si_001.pdf]

Supporting Information

Modulating the Energy Band Structure of Mg-Doped $Sr_{0.5}Pr_{0.5}Fe_{0.2}Mg_{0.2}Ti_{0.6}O_{3-\delta}$ Electrolyte with Boosted Ionic Conductivity and Electrochemical Performance for Solid Oxide Fuel Cells

Sajid Rauf[1*], Muhammad Bilal Hanif[4], Naveed Mushtaq[2,3], Zuhra Tayyab[2], Nasir Ali[5], M.A.K. Yousaf Shah[3], Martin Motola[4], Adil Saleem[6], Muhammad Imran Asghar[2,7**], Rashid Iqbal[8], Changping Yang[2], Wei Xu[1***]

[1]College of Electronics and Information Engineering, Shenzhen University, Guangdong Province, 518000, China.
[2]Hubei Collaborative Innovation Center for Advanced Organic Chemical Materials, Faculty of Physics and Electronic Science, Hubei University, Wuhan, Hubei 430062, P.R. China.
[3]Energy Storage Joint Research Center, School of Energy and Environment, Southeast University, No.2 Si Pai Lou, Nanjing 210096, P.R. China.
[4]Department of Inorganic Chemistry, Faculty of Natural Sciences, Comenius University in Bratislava, 84215 Bratislava, Slovakia.
[5]Zhejiang Province Key Laboratory of Quantum Technology and Devices and Department of Physics and State Key Laboratory of Silicon Materials, Zhejiang University, Hangzhou, 310027, People's Republic of China.
[6]College of Physics and Optoelectronic Engineering, Shenzhen University, Shenzhen 518060, China.
[7]New Energy Technologies Group, Department of Applied Physics, Aalto University School of Science, FI-00076 Aalto, Espoo, Finland.
[8]Institute for Advanced Study, Shenzhen University, Shenzhen 518060, Guangdong, China.

*Corresponding Authors

[1]Corresponding author: Dr. Sajid Rauf sajidrauf@szu.edu.cn

[2]Corresponding author: Prof. Muhammad Imran Asghar imran.asghar@aalto.fi

[3]Corresponding author: Prof. Wei Xu weixu@szu.edu.cn

XRD analysis:

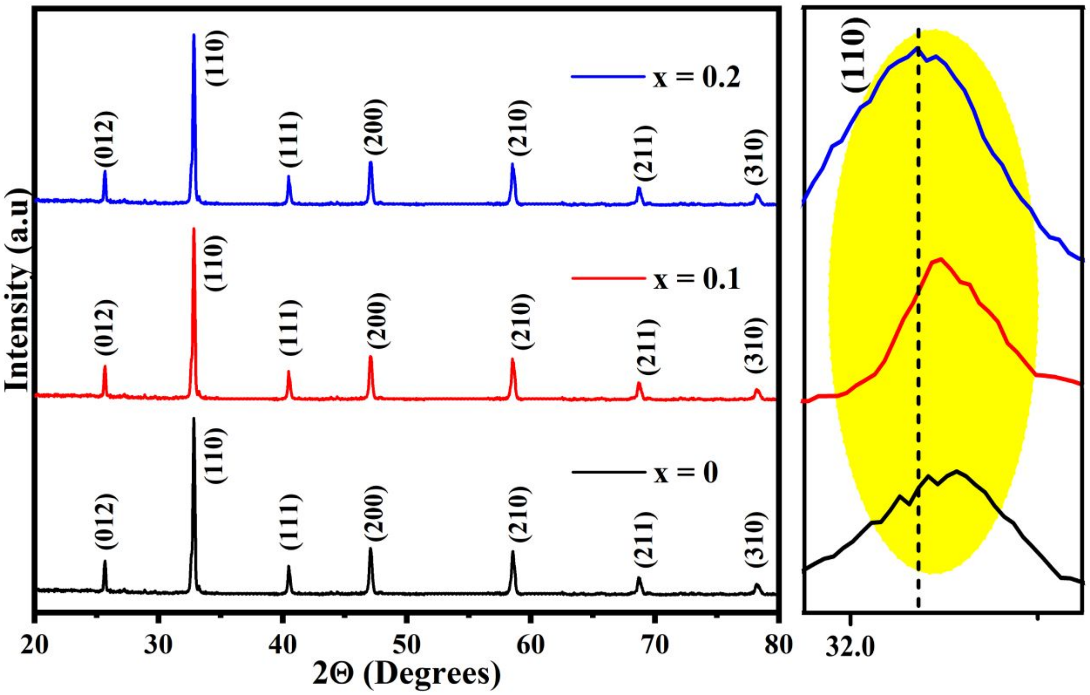

Figure. S1. The x-ray diffraction patterns of $Sr_{0.5}Pr_{0.5}Fe_{0.4-x}Mg_xTi_{0.6}O_{3-\delta}$ (x = 0, 0.1, 0.2) respectively powders and the inset of main peak at (110) plane illustrating the peak shift.

TEM and SEM analyses:

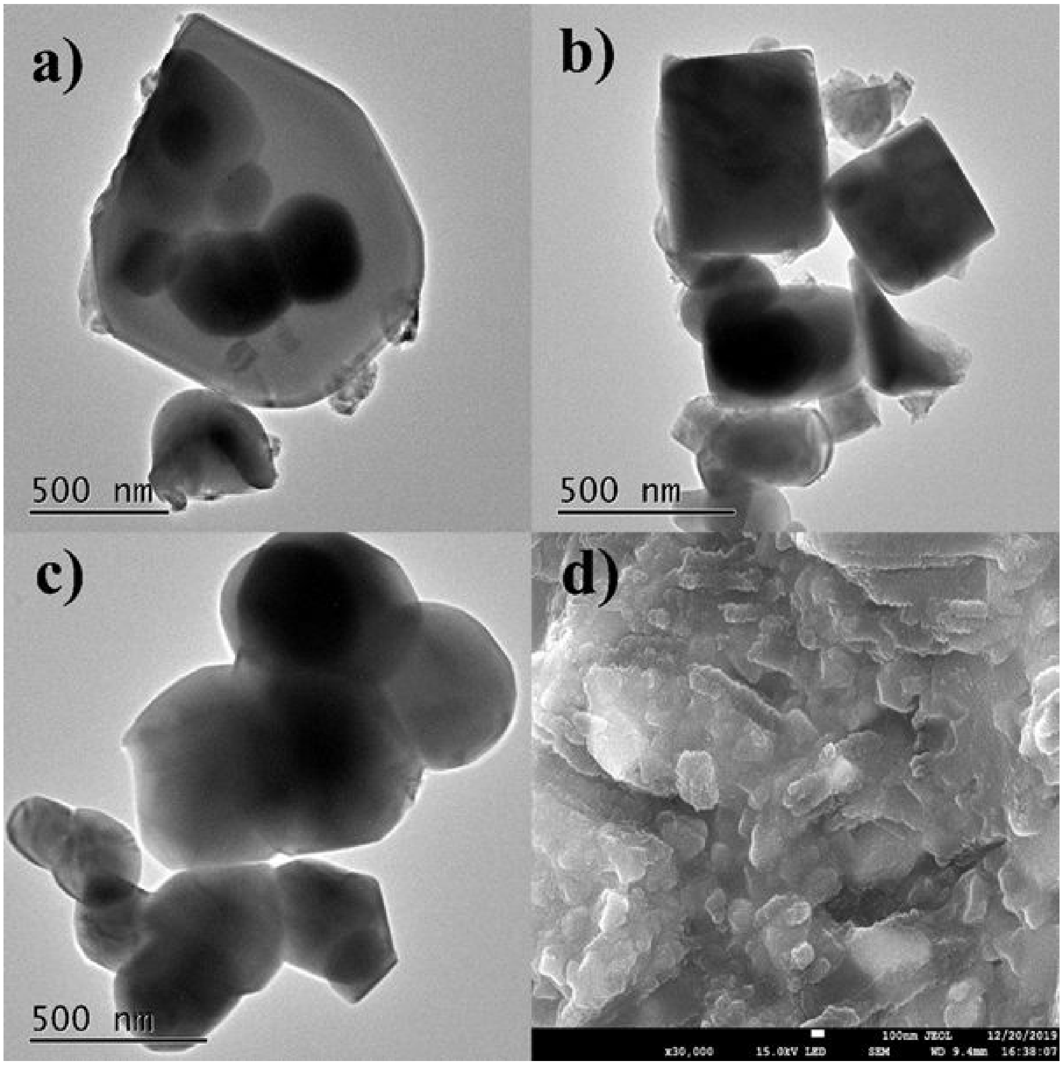

Figure S2. Particles distribution of $Sr_{0.5}Pr_{0.5}Fe_{0.4-x}Mg_xTi_{0.6}O_{3-\delta}$ [x = 0, 0.1, 0.2] revealed by TEM (a-c); and the morphology of the electrolyte $Sr_{0.5}Pr_{0.5}Fe_{0.2}Mg_{0.2}Ti_{0.6}O_{3-\delta}$ (d).

EDS mapping:

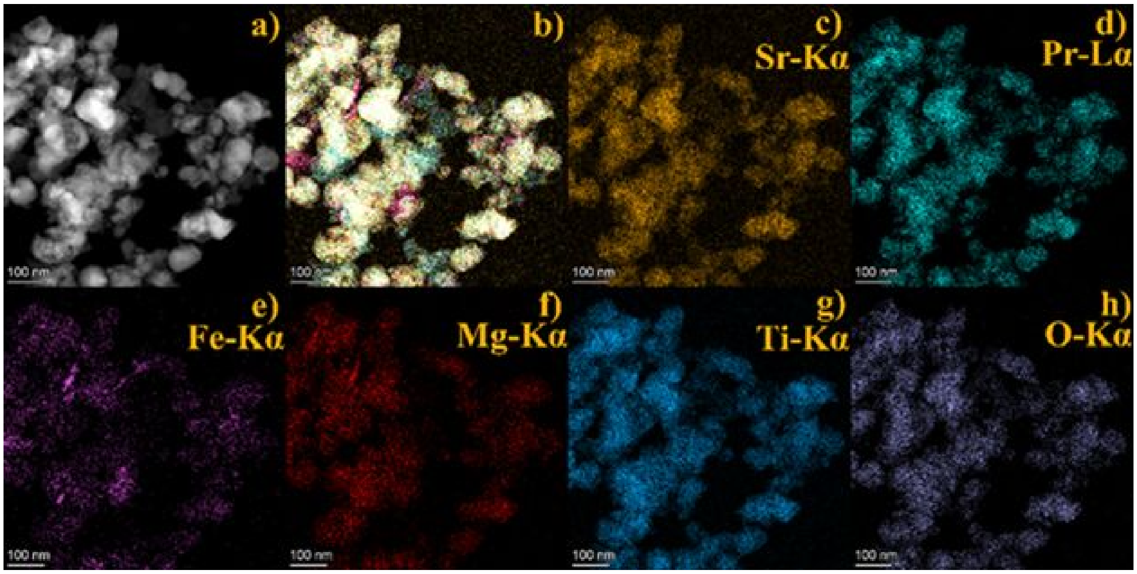

Figure S3. HAADF image and their mixed color image (a-b); and the energy dispersive spectroscopy of each constituent element of $Sr_{0.5}Pr_{0.5}Fe_{0.2}Mg_{0.2}Ti_{0.6}O_{3-\delta}$ (c-h).

XPS analysis:

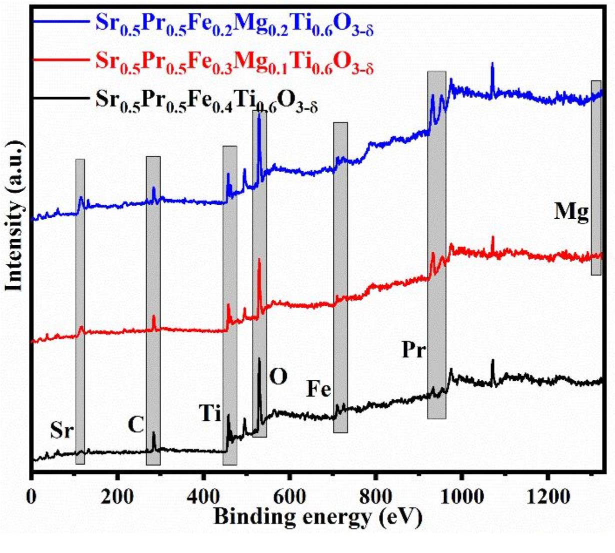

Figure S4. The X-ray photoelectron spectroscopy spectrum of the $Sr_{0.5}Pr_{0.5}Fe_{0.4-x}Mg_xTi_{0.6}O_{3-\delta}$ [x = 0, 0.1, 0.2].

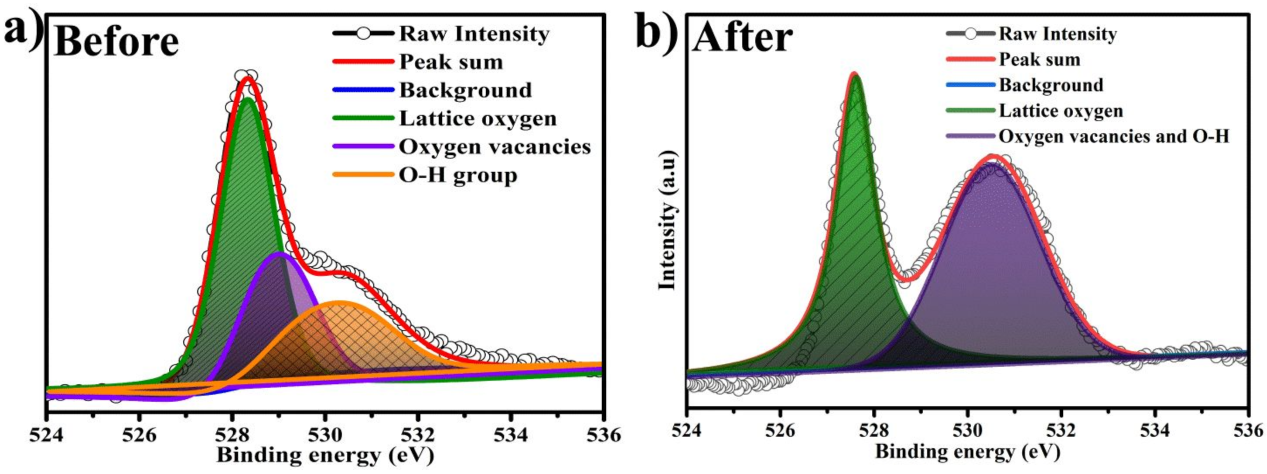

Figure S5. XPS spectra of individual elements of O-1s spectra of $SPFMg_{0.2}T$ of raw powder and after fuel cell performance (scratched from cell utilized in fuel cell performance (a-b).

Fuel cell performance:

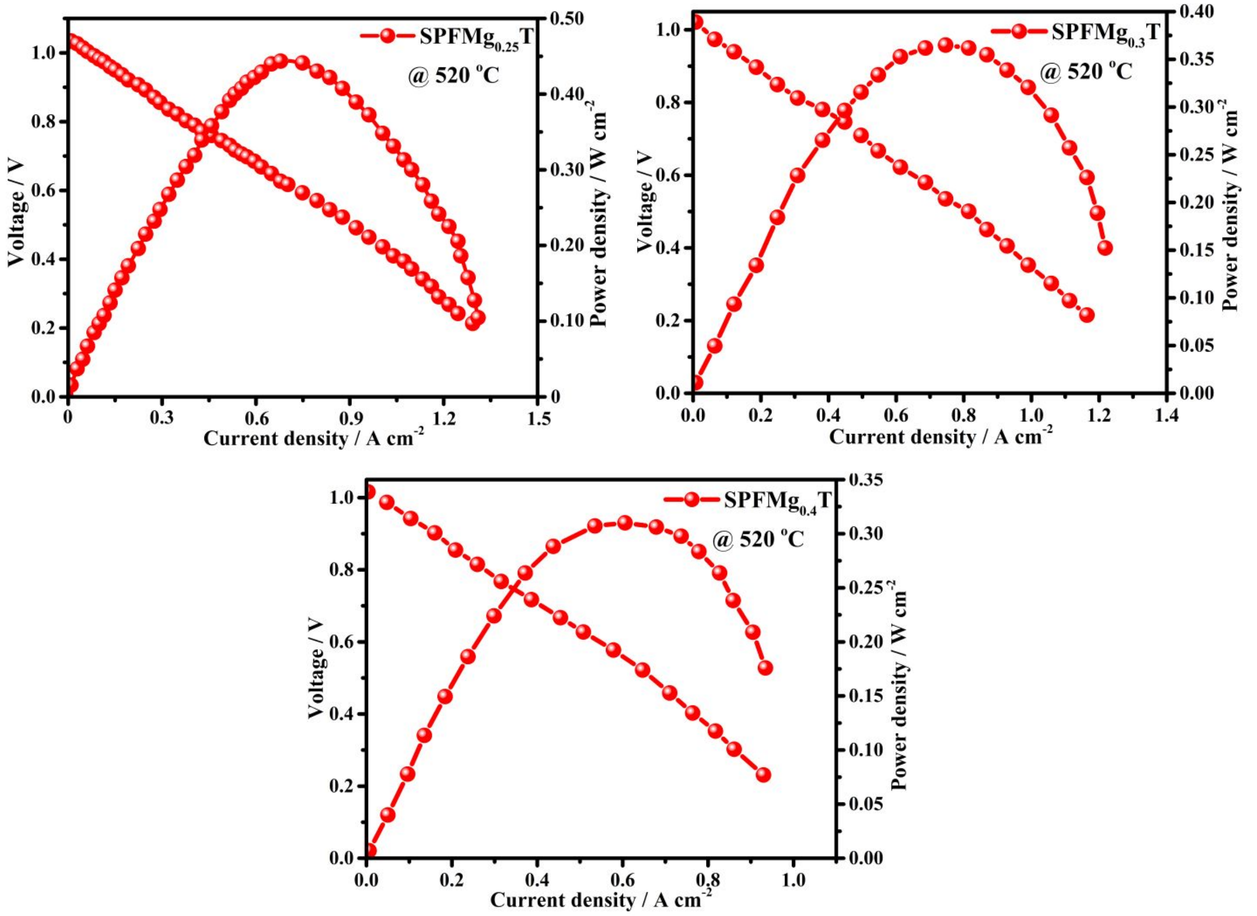

Figure S6. The electrochemical performance in terms of I-V and I-P curves for the fuel cell devices based on $Sr_{0.5}Pr_{0.5}Fe_{0.4-x}Mg_xTi_{0.6}O_{3-\delta}$ [x = 0.25, 0.3, -0.4] electrolyte compositions at operating temperature of 520 ℃ (a-c).

EIS analysis:

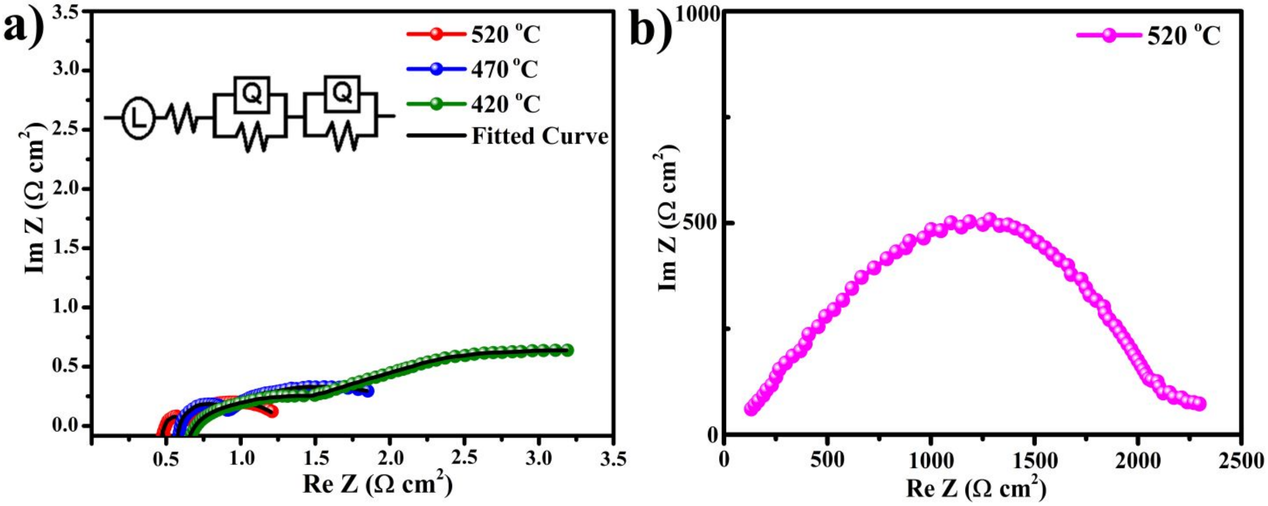

Figure S7. The electrochemical impedance spectroscopy of the $Sr_{0.5}Pr_{0.5}Fe_{0.4}Ti_{0.6}O_{3-\delta}$ electrolyte based fuel cell in H_2/air environment at the temperatures 520 – 420 ℃. (a); the EIS of $Sr_{0.5}Pr_{0.5}Fe_{0.2}Mg_{0.2}Ti_{0.6}O_{3-\delta}$ electrolyte based fuel cell in air/air environment at the temperature 520 ℃ (b).

EELS results:

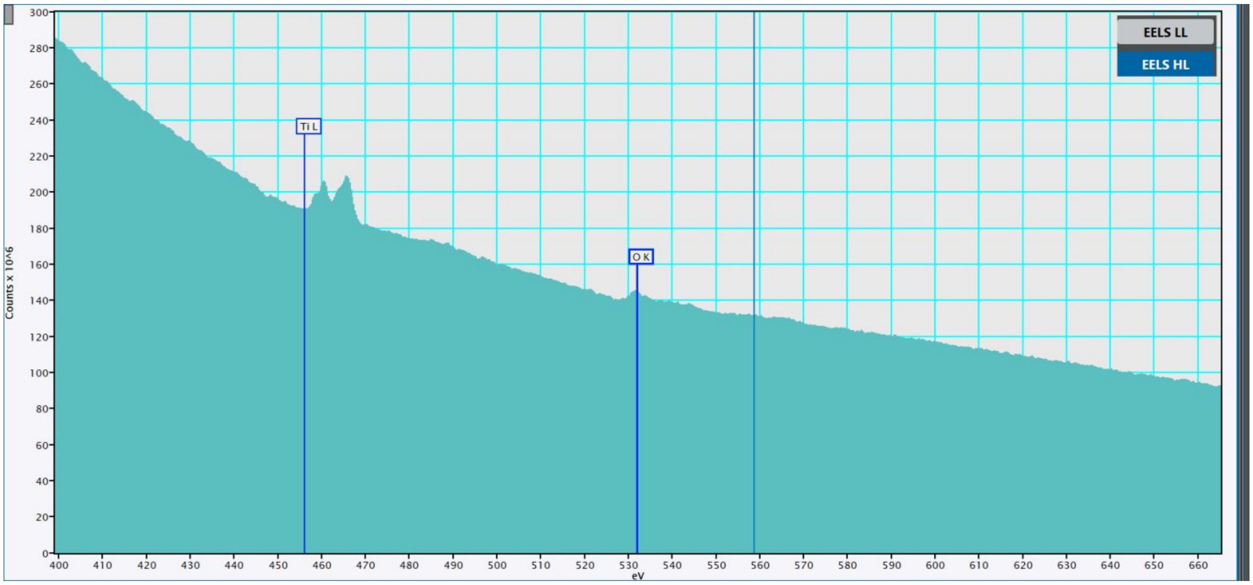

Figure S8. The EELS spectra of Ti and O-edge along the line scan in Fig. 6a

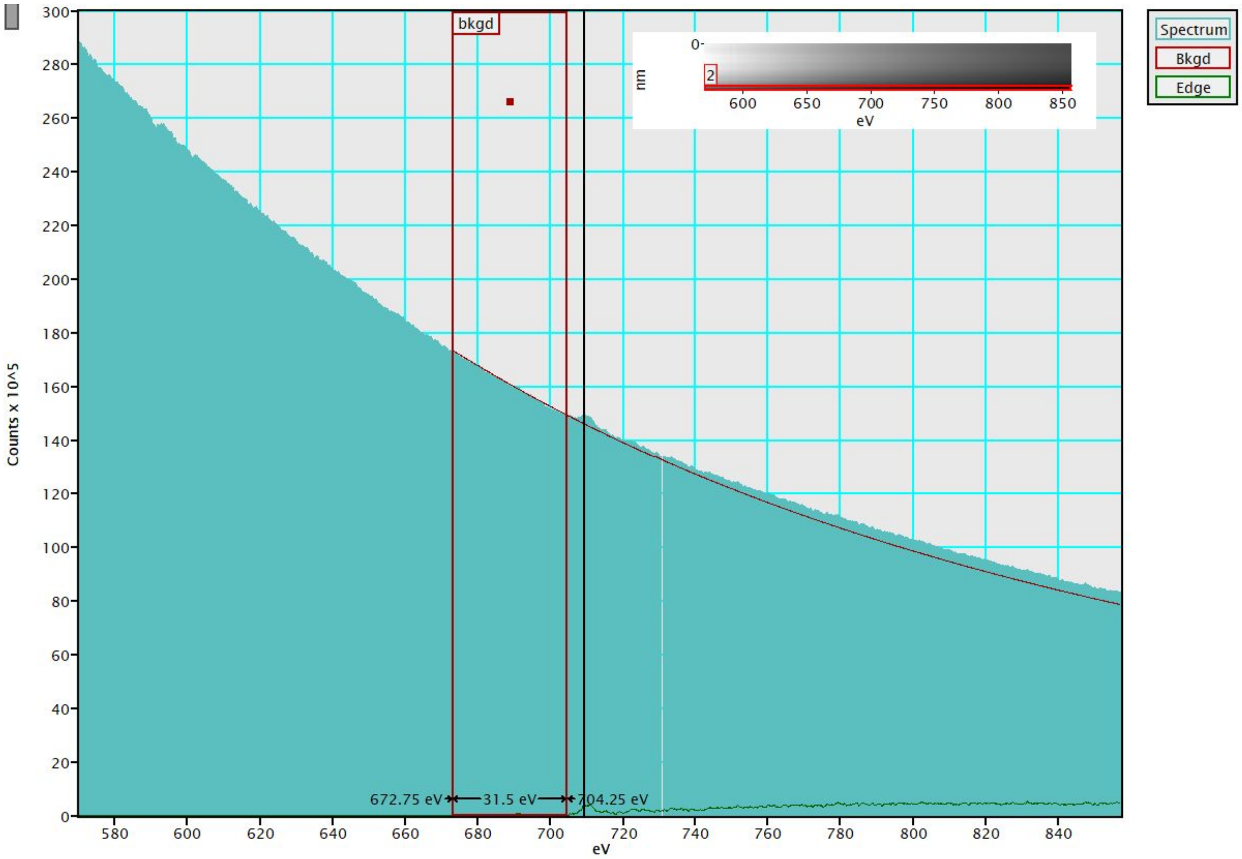

Figure S9. The EELS spectra for Fe along in bulk.

Optical characteristics:

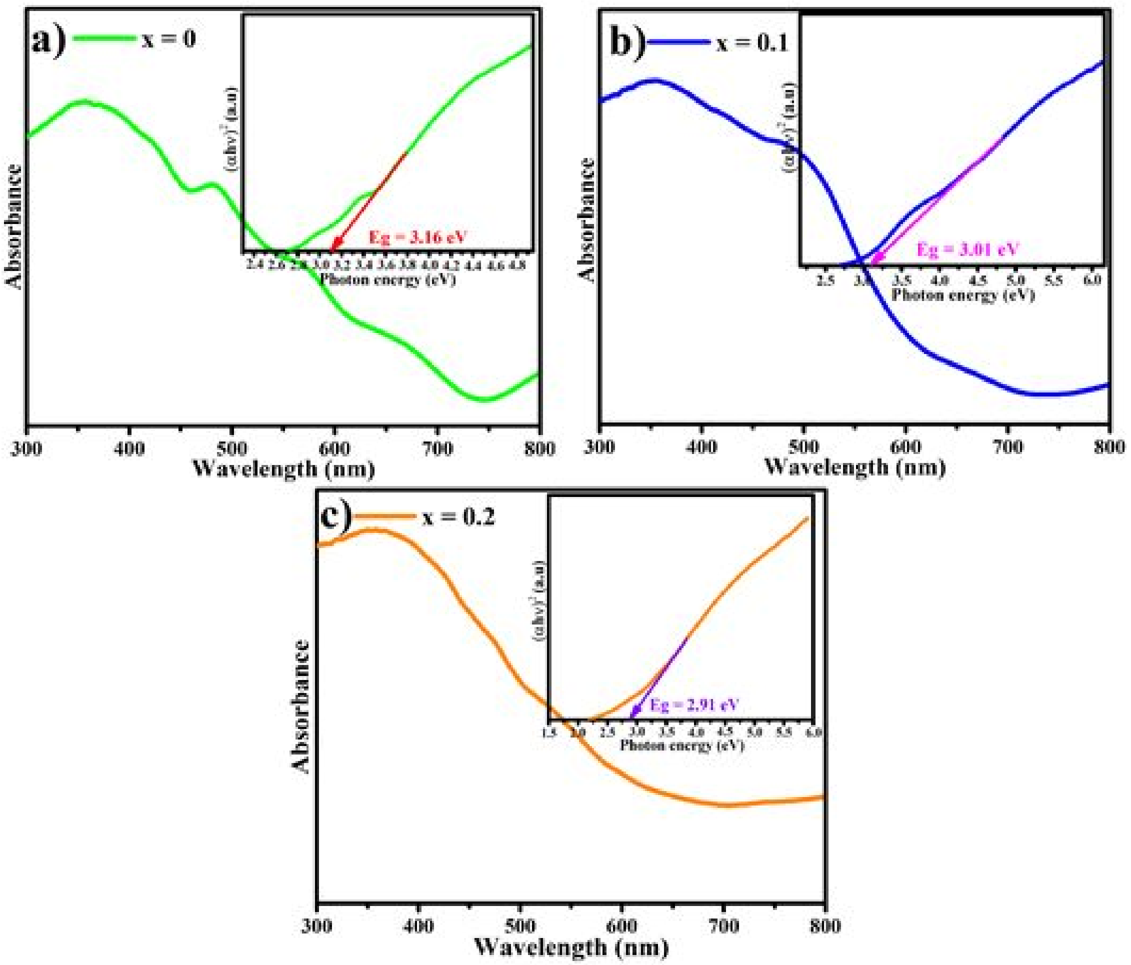

Figure S10. The UV-Vis spectroscopy and energy bandgaps of the $Sr_{0.5}Pr_{0.5}Fe_{0.4-x}Mg_xTi0_{.6}O_{3-\delta}$ (x = 0, 0.1,0.2) (a-c).

The fitting parameters extracted from ZSimpWin with an equivalent circuit of $LR_o(R_1Q_1)(R_2Q_2)$, where R represents a resistance, and Q is the constant phase element (CPE) representing a non-ideal capacitor. The R and Q have the units of Ω cm^2 and F cm^{-2}, respectively. The corresponding characteristic capacitance (Ci) for each process is calculated by:

$$C = \frac{(R_i \times Q_i)^{1/n}}{R_i}.$$

According to the obtain Ci, the semicircles denoted by (R_1Q_1) and (R_2Q_2) can be ascribed to the grain-boundary and electrode polarization processes, respectively. Both the characteristics capacitance of R_1 and R_2 are above 10^{-2} F cm^{-2}, and the corresponding arcs are indexed to the charge transfer process i.e., mass transport resistance, including the molecular gas diffusion, gas

dissociation and adsorption. However, it can be seen that with the reduction of temperature, there is increase of polarization resistance and the capacitance increase, respectively. Since EIS data have two semicircles and Q_1 represent the CPE for the first semicircle and Q_2 for the second semicircle. Furthermore, term "n" belong to the frequency power of Q value. Moreover "n" also denotes the depressed arcs, when value of "n" is close to 1.0, the CPE resembles a capacitor, but the phase angle is not 90°. Here, there are two "n" and both "n" belongs to the frequency power of Q_1 value. 1st "n" is for the semicircle at high frequency and 2nd "n" is for the frequency power of Q_2 value at lower frequency. Moreover, R_0 is ohmic resistance offered by electrolyte, while R_1 and R_2 are charge and mass polarization resistances offered by electrodes and the sum of both R_1 and R_2 is polarization resistances (R_p). As the fuel cell based on electrolyte with high ionic conductivity offer less ohmic resistance (R_0) as well polarization resistances (R_p) are less comparatively.

Table S1. The electrochemical impedance spectra fitted data of $Sr_{0.5}Pr_{0.5}Fe_{0.4}Ti_{0.6}O_{3-\delta}$ (SPFT) electrolyte obtained from Z-Simpwin software at various temperatures 520-420 °C, where R and Q are in **Ω**-cm^2 and $Y_o[(S\text{-}s)^n\ cm^{-2}]$

Sample	Inductance	R_o	R_1	Q_1	n	C_1	R_2	Q_2	n	C_2
**SPFT**										
**520 °C**	1.359E-6	0.49	0.13	0.712	0.975	0.442	0.63	1.242	0.942	0.546
**470 °C**	1.791E-6	0.52	0.42	0.657	0.751	0.565	0.81	0.972	0.700	0.614
**420 °C**	2.256E-6	0.61	0.89	0.626	0.574	0.655	1.75	0.842	0.621	0.715

Table S2. The electrochemical impedance spectra fitted data of $Sr_{0.5}Pr_{0.5}Fe_{0.4-x}Mg_xTi_{0.6}O_{3-\delta}$ [x = 0.1,0.2] electrolyte obtained from Z-Simpwin software at various temperatures 520-420 °C. where R and Q are in **Ω**-cm^2 and $Y_o[(S\text{-}s)^n\ cm^{-2}]$

Sample	Inductance	R_o	R_1	Q_1	n	C_1	R_2	Q_2	n	C_2
**SPFMg$_{0.1}$T**										
**520 °C**	1.782E-7	0.253	0.125	0.578	0.787	0.125	0.578	0.545	0.701	0.213
**470 °C**	1.987E-7	0.354	0.133	0.218	0.654	0.215	0.683	0.341	0.600	0.341
**420 °C**	2.452E-7	0.408	0.192	0.074	0.615	0.287	1.36	0.0124	0.812	0.421
**SPFMg$_{0.2}$T**										
**520 °C**	2.149E-8	0.16	0.06	0.00945	0.852	0.045	0.37	0.0754	0.624	0.081
**470 °C**	2.541E-8	0.20	0.09	0.01585	0.612	0.074	0.60	0.0645	0.545	0.125
**420 °C**	3.246E-8	0.27	0.12	0.05461	0.542	0.094	1.2	0.0101	0.500	0.175
